# Synthetic hyperacetylation of nucleosomal histones†

**DOI:** 10.1039/d0cb00029a

**Published:** 2020-05-19

**Authors:** Hidetoshi Kajino, Tomomi Nagatani, Miku Oi, Tomoya Kujirai, Hitoshi Kurumizaka, Atsuya Nishiyama, Makoto Nakanishi, Kenzo Yamatsugu, Shigehiro A. Kawashima, Motomu Kanai

**Affiliations:** Graduate School of Pharmaceutical Sciences, The University of Tokyo 7-3-1 Hongo Bunkyo-ku Tokyo 113-0033 Japan yamatsugu@mol.f.u-tokyo.ac.jp skawashima@mol.f.u-tokyo.ac.jp kanai@mol.f.u-tokyo.ac.jp; Division of Cancer Cell Biology, The Institute of Medical Science, The University of Tokyo 4-6-1 Shiroganedai Minato-ku Tokyo 108-8639 Japan; Institute for Quantitative Biosciences, The University of Tokyo 1-1-1 Yayoi Bunkyo-ku Tokyo 113-0032 Japan; JST-ERATO, KURUMIZAKA Chromatin Atlas 1-1-1 Yayoi Bunkyo-ku Tokyo 113-0032 Japan

## Abstract

We report combinations of a DMAP-based catalyst and phenyl acetate with optimal electron density as a new chemical system for high-yield, selective synthetic acetylation of histone lysine residues. The utility of this chemical system as a unique biologic tool is demonstrated by applying it to *Xenopus laevis* sperm chromatin.

## Abstract 2

A chemical catalyst system enabling high-yielding and comprehensive lysine acetylation of nucleosomal histones was developed as an epigenetics tool.

## Graphical abstract

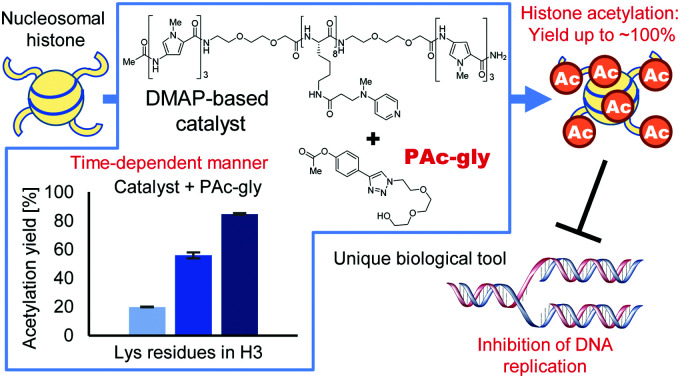

Post-translational modifications of histones, the major proteins in chromatin, play pivotal roles in the epigenome, and abnormalities in these modifications are closely linked to numerous physiological disorders.^1,2^ Chemical tools for manipulating the epigenome hold promise for both therapeutic applications and elucidation of the mechanisms regulating gene expression.^3,4^ We previously developed a chemical system composed of an acylation catalyst (8DMAP, **1**, or 3Py8DMAP3Py (PDP), **3**, Fig. 1a) and an acetyl donor (3NMD8R (NMD: *N*-methoxydiacetamide), **4**) that can bind to chromatin through electrostatic interactions with negatively charged DNA (and in the case of **3**, through hydrogen-bond interactions with DNA base pairs in the minor groove *via* its 3Py units^5^). This binding promotes histone acetylation without reliance on endogenous enzymes (Fig. S1a and b, ESI†).^6^ However, the acetylation yield is only moderate (up to ∼15%)^6^ and thus the utility of this chemical system has been limited. The low yield is partly due to the high reactivity of 3NMD8R: the donor spontaneously hydrolyzes in buffer within several hours (Fig. S1c, ESI†), and also is consumed through undesired background reactions with non-histone proteins in a catalyst-independent manner (Fig. S1d, ESI†). We therefore aimed to develop a new acetyl donor that is less reactive and is activated in the presence of catalysts.

**Fig. 1:**
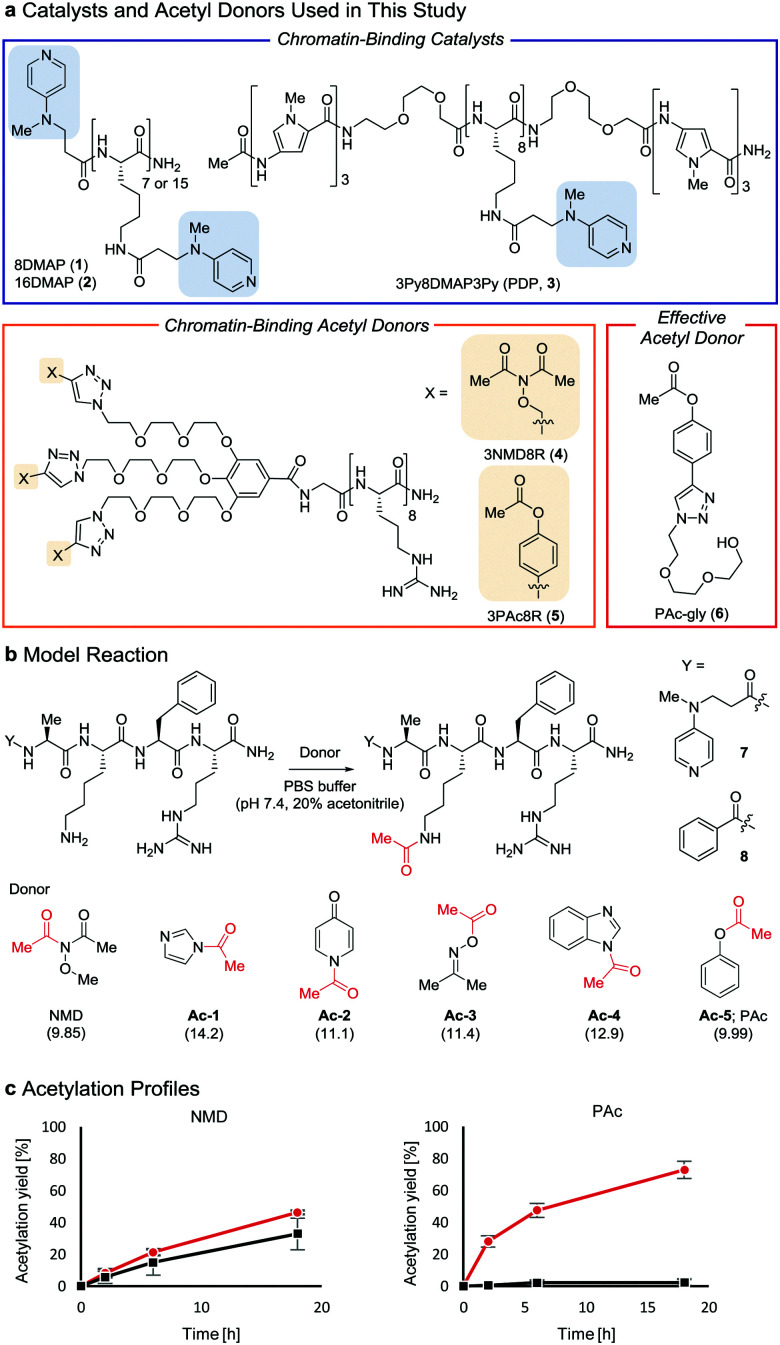
Properties of acetyl donors. (a) Chemical structures of the catalysts and acetyl donors. (b) Screening of acetyl donors. The p*K*_a_ value of each leaving group conjugate acid is shown in parentheses. (c) Comparison of NMD and **Ac-5** using **7** (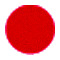) and **8** (■) at 30 °C. The average and SD (bars) are indicated (*n* = 3 independent experiments).

Two lysine-containing peptides with or without the DMAP moiety (DMAP-AKFR, **7**, and Bz-AKFR, **8**) were prepared as model systems for directed catalysis^7^ and catalyst-independent control, respectively. These peptides were reacted with five acetyl donor candidates (**Ac-1**–**5**, Fig. 1b) selected for the lower acidity of their leaving group conjugate acid compared to *N*-methoxyacetamide.^8–11^ When **Ac-1** or **Ac-2** was used, rapid acetylation of both **7** and **8** was observed in 2 h and then acetylation stopped, suggesting that **Ac-1** and **Ac-2** were hydrolyzed within 2 h (Fig. S1e, ESI†). When **Ac-3**, **Ac-4**, or **Ac-5** was used, acetylation of **8** proceeded in low yield (<15%), suggesting that the background reactivity of these donors was much lower than that of NMD (Fig. 1c and Fig. S1e, ESI†). Acetylation of **7** containing the DMAP moiety, however, efficiently proceeded when **Ac-4** or **Ac-5** was used, but not **Ac-3** (Fig. 1c and Fig. S1e, ESI†). The greatest rate-enhancement by the DMAP moiety was observed in **Ac-5**: the relative initial reaction rates between **7** and **8** were 86-fold using **Ac-5** and 1.5-fold using NMD. Based on these data, we concluded that **Ac-5** (*i.e.*, phenyl acetate (PAc)) is the best acetyl donor.

We rationalized the observed favorable properties of PAc in DMAP-catalyzed lysine acetylation using DFT calculations (for the overall energy diagram, see Fig. S2, ESI†). Protonated methylamine rather than lysine was used as a model substrate in the theoretical studies. The rate-determining step was the generation of the acetyl pyridinium ion (from **IMPAc2** to **IMPAc5**, Fig. 2a). Of note is the identified transition state of this step, **TSPAc4**, which was stabilized by formation of a six-membered ring comprising acetylated DMAP, the protonated amine, and the leaving phenoxide ion through electrostatic interactions and hydrogen bonds. This stabilization simultaneously promoted both formation of the acetyl pyridinium electrophile and the deprotonated amine nucleophile. The concerted generation of the nucleophile/electrophile pair in close proximity would be beneficial to the total kinetics.

**Fig. 2:**
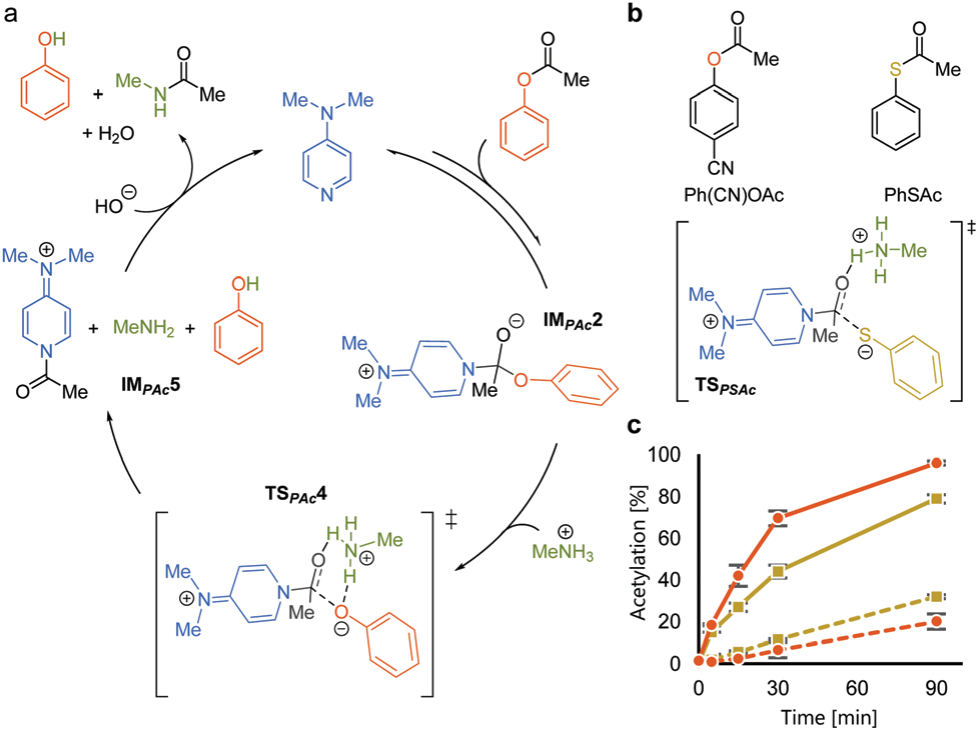
Mechanistic analyses of DMAP-catalyzed acetylation with PAc. (a) A plausible catalytic cycle based on DFT calculations. (b) Comparison between phenyl acetate and phenyl thioacetate. (c) Reaction profiles of the acetylation of **7** (solid lines) and **8** (dotted lines) using Ph(CN)OAc (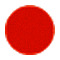) and PhSAc (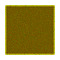) at 30 °C.

To experimentally investigate the importance of the six-membered transition state (**TSPAc4**), we used *S*-phenyl thioacetate (PhSAc, Fig. 2b) as an acetyl donor. In this case, the leaving group was less basic than the phenoxide anion and thus would not form the six-membered transition state through interaction with the protonated amine (**TSPSAc**). Instead of PAc, Ph(CN)OAc was used for comparison, since its catalyst-independent reactivity was nearly comparable to that of PhSAc (dotted lines in Fig. 2c). As expected, **7** was more efficiently acetylated by Ph(CN)OAc than PhSAc (solid lines in Fig. 2c), suggesting the importance of the basicity of the phenoxide anion in DMAP-catalyzed lysine acetylation.

Next, we synthesized 3PAc8R (**5**) as a chromatin-binding acetyl donor (Fig. 1a). We compared the susceptibility to hydrolysis of **5** and **4** in buffer (Fig. S3a, ESI†). Less than 40% of **4** remained after 6 h compared with approximately 80% of **5**, indicating that **5** was more resistant to unproductive hydrolysis than **4**. Then, we compared the reactivity and selectivity of **5** and **4** using a mixture of recombinant nucleosomes and HeLa cell extract as substrate (Fig. 3a). In the absence of a catalyst, **4** acetylated both histone and non-histone proteins in a concentration-dependent manner, whereas no background lysine acetylation of non-histone proteins by **5** was detected in the same concentration range. The combination of the PDP catalyst (**3**) and **5** selectively promoted the acetylation of histones (Fig. 3b), showing that **5** was efficiently activated by **3** to promote histone-selective acetylation, and that hydrolysis and catalyst-independent background acetylation were suppressed.

**Fig. 3:**
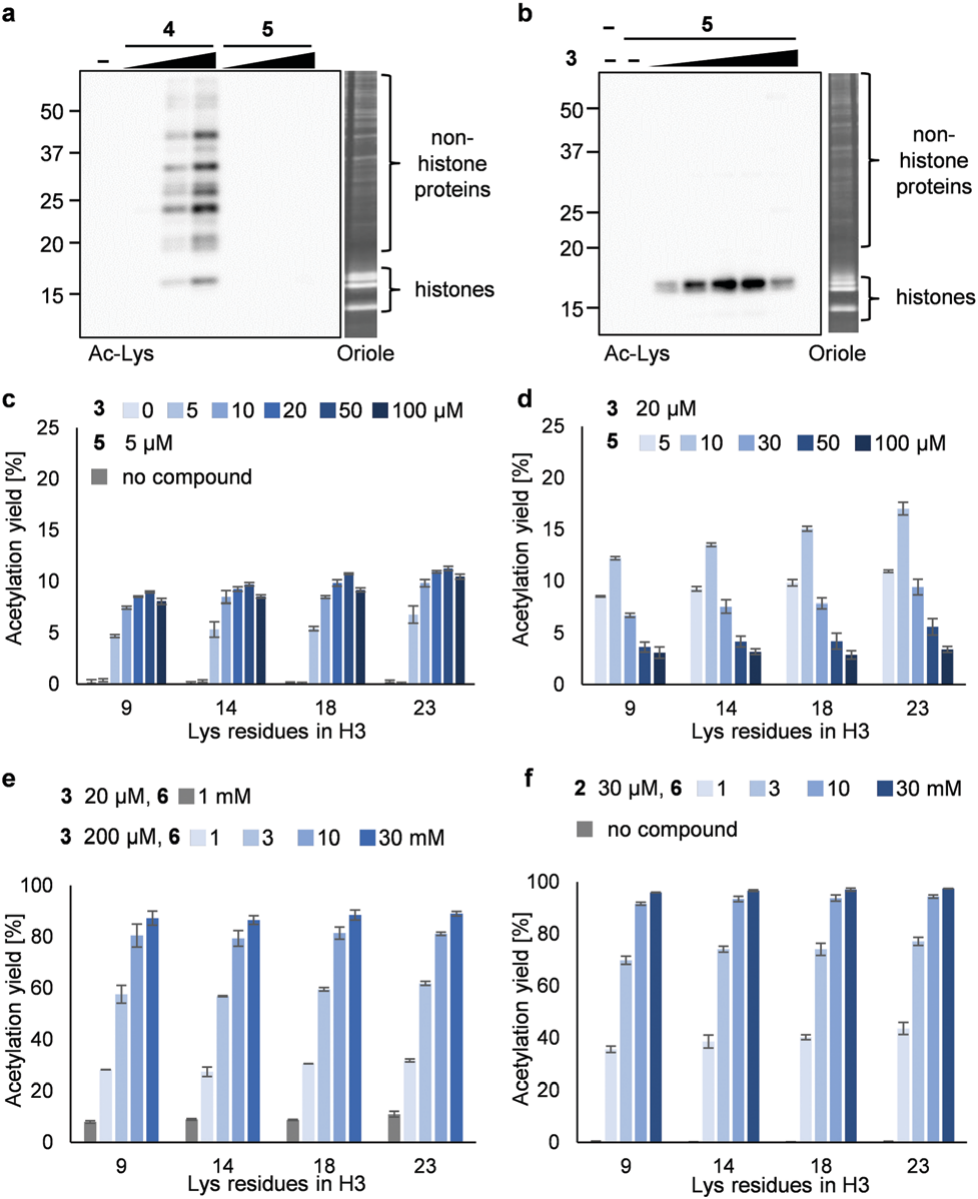
Acetylation of a mixture of recombinant nucleosomes (0.2 μM as DNA concentration) and HeLa cell extract (non-histone proteins) at 25 °C for 3 h. (a) Background histone acetylation by **4** (2, 5, and 10 μM) or **5** (2, 5, and 10 μM). (b) Catalyzed histone acetylation by **3** (5, 10, 20, 50, and 100 μM) and **5** (5 μM). In (a and b), acetylated lysines were detected by immunoblotting using an anti-Ac-Lys antibody. Proteins were visualized by Oriole staining. (c) Yield of histone acetylation by **3** (5, 10, 20, 50, and 100 μM) and **5** (5 μM). (d) Yield of histone acetylation by **3** (20 μM) and **5** (5, 10, 30, 50, and 100 μM). (e) Yield of histone acetylation by **3** (20 μM) and **6** (1 mM) or **3** (200 μM) and **6** (1, 3, 10, and 30 mM). (f) Yield of histone acetylation by **2** (30 μM) and **6** (1, 3, 10, and 30 mM). In (c–f), the yield of acetylated lysines of the H3 tail was determined by LC-MS/MS analysis. The average and error range (bars) are indicated (*n* = 2 independent experiments).

We then quantified the acetylation yield of lysines in the H3 tail by LC-MS/MS.^6^ Yields of up to ∼10% were obtained using 5 μM **5** and 5–20 μM **3** (Fig. 3c); higher concentrations of **3** did not increase the yield. The yield increased slightly to ∼15% when 20 μM **3** and 5–10 μM **5** were used (Fig. 3d). Unexpectedly, however, further addition of **5** decreased the yield dramatically (Fig. 3d). Since the binding modes of **3** and **5** to nucleosomes are identical (*i.e.*, electrostatic interactions with DNA), the donor and catalyst may compete in substrate binding, resulting in moderate yield. This hypothesis was supported by electrophoretic mobility shift assay, showing that the affinity of **5** for nucleosomes was higher than that of **3** (Fig. S3b, ESI†).

Because the catalyst-independent background lysine acetylation hardly proceeded with PAc, we expected that a phenyl acetate donor lacking the nucleosome-binding 8R motif could be activated by **3** bound to nucleosomes, promoting histone-selective acetylation without competition between the donor and the catalyst in substrate binding. We therefore synthesized PAc-gly (**6**), which has a triethylene glycol moiety for water solubility, as a nucleosome-non-binding acetyl donor (Fig. 1a). Recombinant nucleosomes were hardly acetylated even when 30 mM **6** was used without a catalyst (Fig. S3c, ESI†), while the addition of catalyst **3** (20 or 200 μM) markedly promoted histone acetylation in a **6** concentration-dependent manner (Fig. 3e (H3 tail) and Fig. S3d (H3 fold domain), ESI†). A western blot analysis using an anti-Ac-Lys antibody showed that the band corresponding to histones (especially H3) was detected by combining **6** with **3**, suggesting a histone-selective acetylation reaction (Fig. S3e, ESI†). When 200 μM **3** and 30 mM **6** were used, the acetylation yield of lysines on the H3 tail was enhanced up to almost 90% (Fig. 3e). We also tested another nucleosome-binding catalyst, 16DMAP (**2**, Fig. 1a) and found that a combination of 30 μM **2** with 30 mM **6** promoted H3 acetylation almost to completion (Fig. 3f (H3 tail) and Fig. S3f (H3 fold domain), ESI†).

This chemical catalyst system can be used as a unique tool to study cell cycle events by manipulating the histone acetylation status of biologically relevant chromatin. We chose *Xenopus laevis* sperm chromatin (XSC) as a substrate.^12^ XSC contains low levels of H3 acetylation (up to ∼10%, Fig. S4a, ESI†). A major histone acetyltransferase (HAT), p300,^13^ was unable to promote the acetylation of H3 in XSC (Fig. S4a, ESI†), suggesting that HATs were not suitable to manipulate the histone acetylation status of XSC. In contrast, the new chemical catalyst system efficiently acetylated H3 in XSC (Fig. S4b, ESI†). Importantly, by changing the reaction time, we could synthesize XSC with a variety of histone acetylation levels (10–80%, Fig. S4b, ESI†).

Highly acetylated XSC (reaction time = 7 h, ∼80% yield) was added to *Xenopus* egg extracts,^14^ and the cell cycle events were studied. Interestingly, there was little DNA replication until around 2 h after addition (Fig. 4a). XSC treated with only PAc-gly **6** or 16DMAP **2** served as controls and did not significantly inhibit DNA replication compared with untreated XSC (Fig. 4a). We next studied the correlation between the acetylation level of XSC and the retardation of DNA replication by preparing XSC with variable acetylation levels according to the reaction time (∼10% (0.5 h), ∼20% (1 h), or ∼60% (3 h), Fig. S4b, ESI†). Partial inhibition of DNA replication was observed even when using ∼10% acetylated XSC (Fig. 4b). XSC with a higher acetylation level inhibited DNA replication to a higher extent (Fig. 4b). We investigated the phenotype in more detail by examining the DNA replication status and chromatin morphology by fluorescence microscopy. In the untreated control samples, Cy3-dCTP-containing round nuclei were observed within 2 h, indicating correct nuclear formation and DNA replication (Fig. 4c). However, when ∼80% (7 h) acetylated XSC was used, Cy3 signals or round nuclei were hardly observed (Fig. 4c), suggesting that synthetic histone acetylation inhibited proper nuclear formation.

**Fig. 4:**
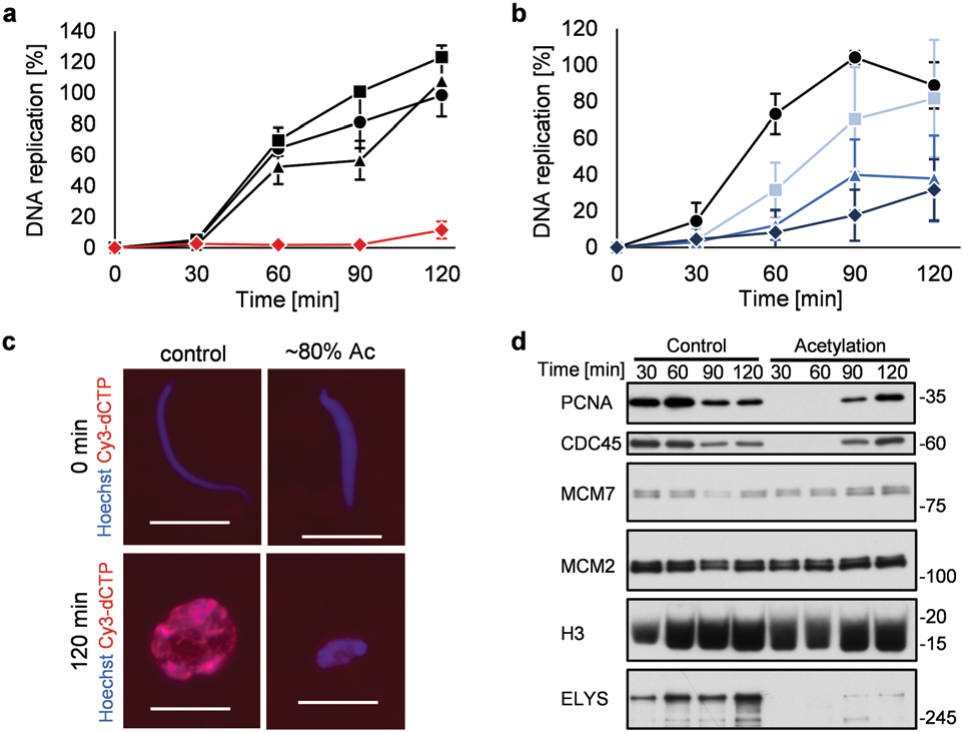
Synthetic histone acetylation inhibits DNA replication. (a and b) XSC (20 000 sperm per μL) was treated with **2** (30 μM) and **6** (30 mM) for (a) 7 h or (b) the indicated time at 25 °C. After the reaction, control or acetylated XSC was incubated with interphase egg extracts containing radiolabeled [α-^32^P]-dCTP. The DNA replication kinetics in egg extracts was assessed by the incorporation of radioactivity, determined using a scintillation counter. Data shown are the average and SD (bars) from (a) three or (b) five independent experiments. (a) ●: control; ■: **2** only; ▲: **6** only; 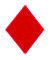: **2** + **6**. (b) ●: control. Reaction time = 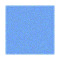: 0.5 h; 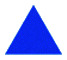: 1 h; 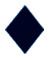: 3 h. (c) and (d) XSC (20 000 sperm per μL) was treated with **2** (30 μM) and **6** (30 mM) for 7 h. (c) Control or acetylated XSC was incubated with interphase egg extracts containing Cy3-dCTP. After incubation, the samples were fixed and DNA was visualized with Hoechst 33258 (blue image). DNA replication was monitored as the incorporation of Cy3-dCTP (red image) into DNA. The scale bar represents 20 μm. (d) Control or acetylated XSC was incubated with interphase egg extracts. Chromatin was isolated from the egg extracts at the indicated times and the chromatin samples were analyzed by immunoblotting using the indicated antibodies.

We gained insight into which DNA replication processes are affected by histone acetylation by examining the chromatin binding of several DNA replication factors by immunoblotting (Fig. 4d). Chromatin binding of MCM2 and MCM7,^15^ which are required for formation of the pre-replication complex (pre-RC),^16^ was comparable between the control and acetylated chromatin, suggesting that pre-RC formation was unaffected by histone acetylation. In contrast, chromatin binding of CDC45,^17^ which is required for formation of the pre-initiation complex (pre-IC),^16^ was significantly delayed in the acetylated samples. Furthermore, chromatin binding of PCNA, a processivity factor for DNA polymerases,^18^ was also delayed.

These results suggest that histone acetylation inhibited pre-IC formation. Nuclear transport of several factors such as protein kinases through the nuclear pore complex (NPC) plays a critical role in pre-IC formation. NPC formation is initiated by ELYS, the chromatin-associated nucleoporin (NUP).^19^ Then, ELYS recruits a complex of NUPs to coordinate nuclear assembly during DNA replication.^20^ We therefore investigated whether chromatin binding of ELYS was affected in the acetylated samples and intriguingly found that chromatin binding of ELYS was significantly reduced on acetylated chromatin (Fig. 4d). Taken together, our data suggest that the inhibition of DNA replication associated with synthetic histone acetylation occurs by retardation of ELYS–chromatin binding, which may cause incomplete NPC formation and abnormalities in the nuclear transport of DNA replication factors such as CDC45 and PCNA (Fig. S4c, ESI†).

In this study, we developed the new acetyl donor PAc-gly for highly effective synthetic histone acetylation. While PAc-gly itself hardly reacted with proteins or underwent hydrolysis, DMAP-based chromatin-binding catalysts efficiently activate PAc-gly, probably *via* concerted generation of the acetyl pyridinium electrophile and the deprotonated lysine nucleophile, promoting histone acetylation in up to almost 100% yield. Using this chemical catalyst system, we found that synthetic acetylation of *Xenopus* chromatin prevents DNA replication in *Xenopus* egg extracts, likely by inhibiting chromatin binding of ELYS. Further analysis of the phenotypes may provide new insights into the role of histone acetylation in cell cycle events involving chromatin.

## Conflicts of interest

There are no conflicts to declare.

## Supplementary Material

CB-001-D0CB00029A-s001
