# Supplementary material for: Synthetic hyperacetylation of nucleosomal histones
Source: RSC Chem Biol. 2020 May 19;1(2):56–9. doi: 10.1039/d0cb00029a (PMC8341002; doi:10.1039/d0cb00029a)
Supplement: CB-001-D0CB00029A-s001 [file CB-001-D0CB00029A-s001.pdf]

Electronic Supplementary Material (ESI) for RSC Chemical Biology.
This journal is © The Royal Society of Chemistry 2020

Supporting Information

Synthetic Hyperacetylation of Nucleosomal Histones

Hidetoshi Kajino,[a] Tomomi Nagatani,[b] Miku Oi,[a] Tomoya Kujirai,[c,d] Hitoshi Kurumizaka,[c,d] Atsuya Nishiyama,[b] Makoto Nakanishi,[b] Kenzo Yamatsugu,[a]* Shigehiro A. Kawashima,[a]* & Motomu Kanai[a]*

[a]Graduate School of Pharmaceutical Sciences, The University of Tokyo, 7-3-1 Hongo, Bunkyo-ku, Tokyo 113-0033, Japan

[b]Division of cancer cell biology, The institute of Medical Science, The University of Tokyo, 4-6-1 Shiroganedai, Minato-ku, Tokyo 108-8639, Japan

[c]Institute for Quantitative Biosciences, The University of Tokyo, 1-1-1 Yayoi, Bunkyo-ku, Tokyo, 113-0032, Japan

[d]JST-ERATO, KURUMIZAKA Chromatin Atlas, 1-1-1 Yayoi, Bunkyo-ku, Tokyo, 113-0032, Japan

Correspondence to
Motomu Kanai (kanai@mol.f.u-tokyo.ac.jp)
Shigehiro A. Kawashima (skawashima@mol.f.u-tokyo.ac.jp)
Kenzo Yamatsugu (yamatsugu@mol.f.u-tokyo.ac.jp)

Contents

1. Supporting figures: S1–S4 S1

2. Experimental procedures

General S6

Materials S6

Synthetic procedures S7

Chemical experiments S12

Biochemical experiments S13

Computational general information S14

Cartesian coordinates and energies S15

3. References S24

4. Spectral data S26

1. Supporting figures

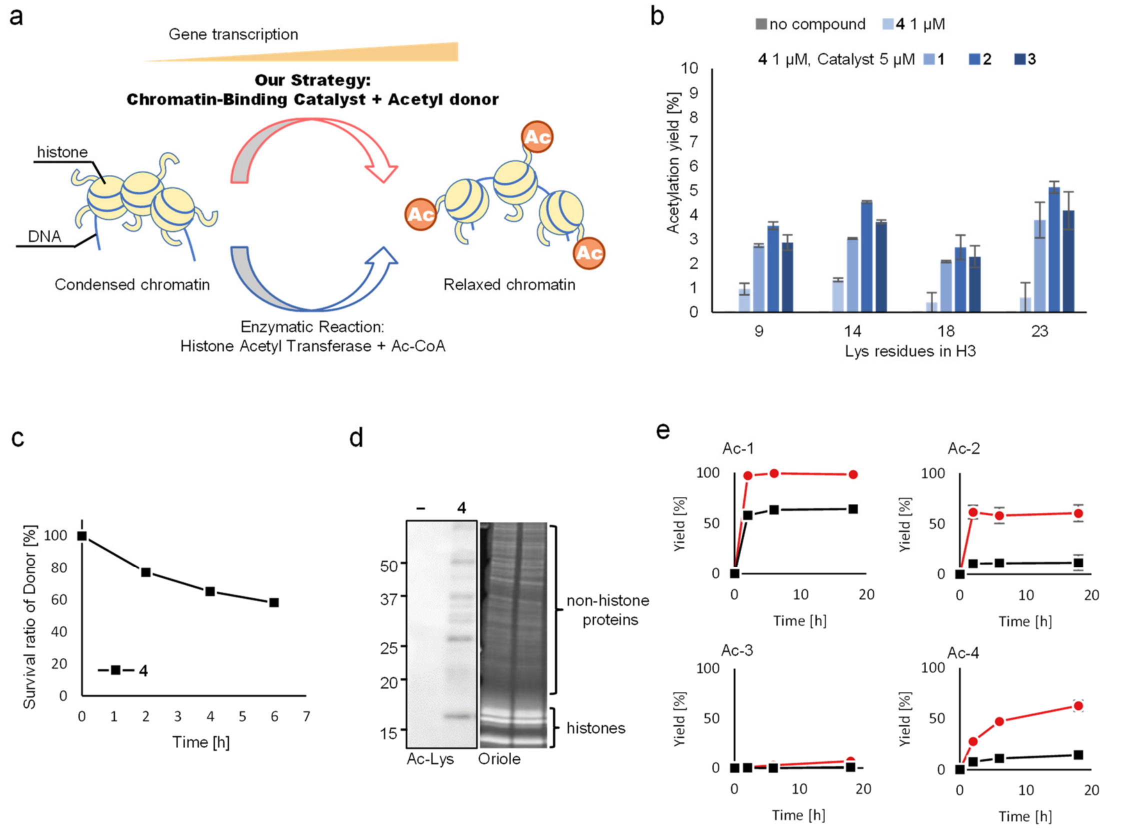

Figure S1. (a) Schematic for synthetic histone acetylation by an chemical catalyst system. (b) Yield of histone acetylation of recombinant nucleosomes (0.2 μM as DNA concentration) by **4** (1 μM) and/or **1**, **2** or **3** (5 μM) for 3 h at 25 °C. The stoichiometry of the acetylated lysines of H3 tail was analyzed by LC-MS/MS. The average and error bars are indicated (twice measurement in the same experiments). (c) A hydrolysis profile of **4** (black ■) at 25 °C. (d) Histone acetylation of recombinant nucleosomes (0.2 μM as DNA concentration) in the presence of HeLa cell extract (non-histone proteins) by **4** (5 μM) for 3 h at 25 °C. Acetylated lysines were detected by immunoblotting using an anti-Ac-Lys antibody. Proteins were visualized by Oriole staining. (e) Acetylation profiles of **Ac-1**–**4** using **7** (●) and **8** (■) at 30 °C. The average and SD (bars) are indicated (n = 3 independent experiments).

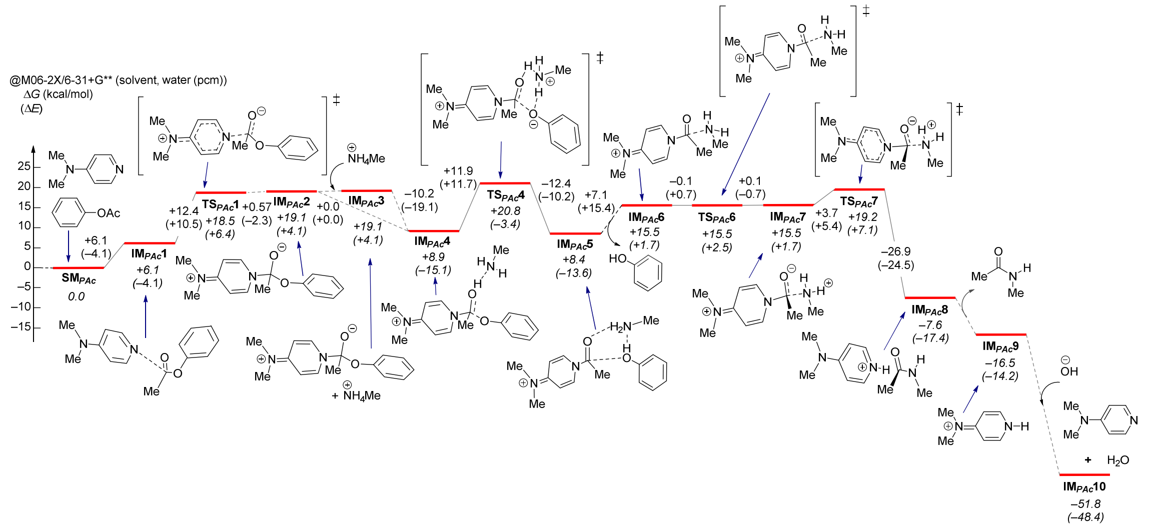

Figure S2. Energy profile of the DMAP-catalyzed acetylation of methylamine with phenyl acetate, as calculated at the M06-2X/6-31+G** (solvent: water (pcm)).

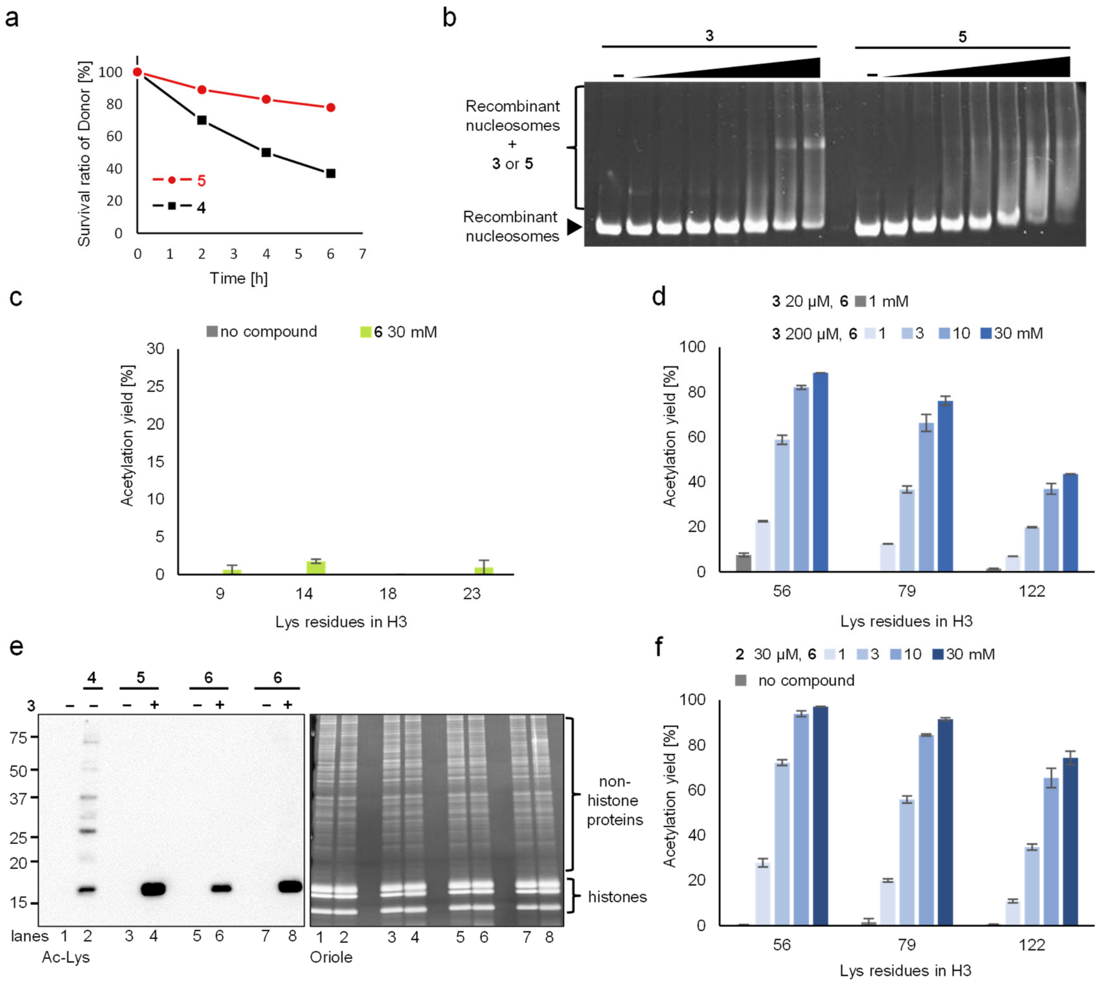

Figure S3. (a) Hydrolysis profiles of **4** (black ■) and **5** (red ●) at 37 °C. (b) Electrophoretic mobility shift assay of recombinant nucleosomes in the presence of **3** or **5**. Recombinant nucleosomes (0.2 μM) were incubated with **3** (0.5, 1, 1.5, 2, 3, 4, 5 μM) or **5** (0.5, 1, 1.5, 2, 3, 4, 5 μM), and were analyzed by 6% non-denaturing polyacrylamide gel electrophoresis in 0.2× TBE buffer. The bands were visualized by ethidium bromide staining. The position of recombinant nucleosomes is shown. (c) Yield of histone acetylation of recombinant nucleosomes (0.2 μM as DNA conc.) by **6** (30 mM) for 3 h at 25 °C. (d) Yield of histone acetylation by **3** (20 μM) and **6** (1 mM) or **3** (200 μM) and **6** (1, 3, 10, 30 mM). (e) Histone acetylation of recombinant nucleosomes (0.2 μM as DNA conc.) in the presence of HeLa cell extract (non-histone proteins) by **4** (10 μM); lane 2, **3** (20 μM) and **5** (5 μM); lane 4, **3** (20 μM) and **6** (0.5 mM); lane 6, or **3** (20 μM) and **6** (1 mM); lane 8 for 3 h at 25 °C. Acetylated lysines were detected by immunoblotting using an anti-Ac-Lys antibody. Proteins were visualized by Oriole staining. (f) Yield of histone acetylation by **2** (30 μM) and **6** (1, 3, 10, 30 mM). In (d) and (f), the yield of acetylated lysines of H3 fold domain was determined by LC-MS/MS analysis. The average and error range (bars) are indicated (n = 2 independent experiments).

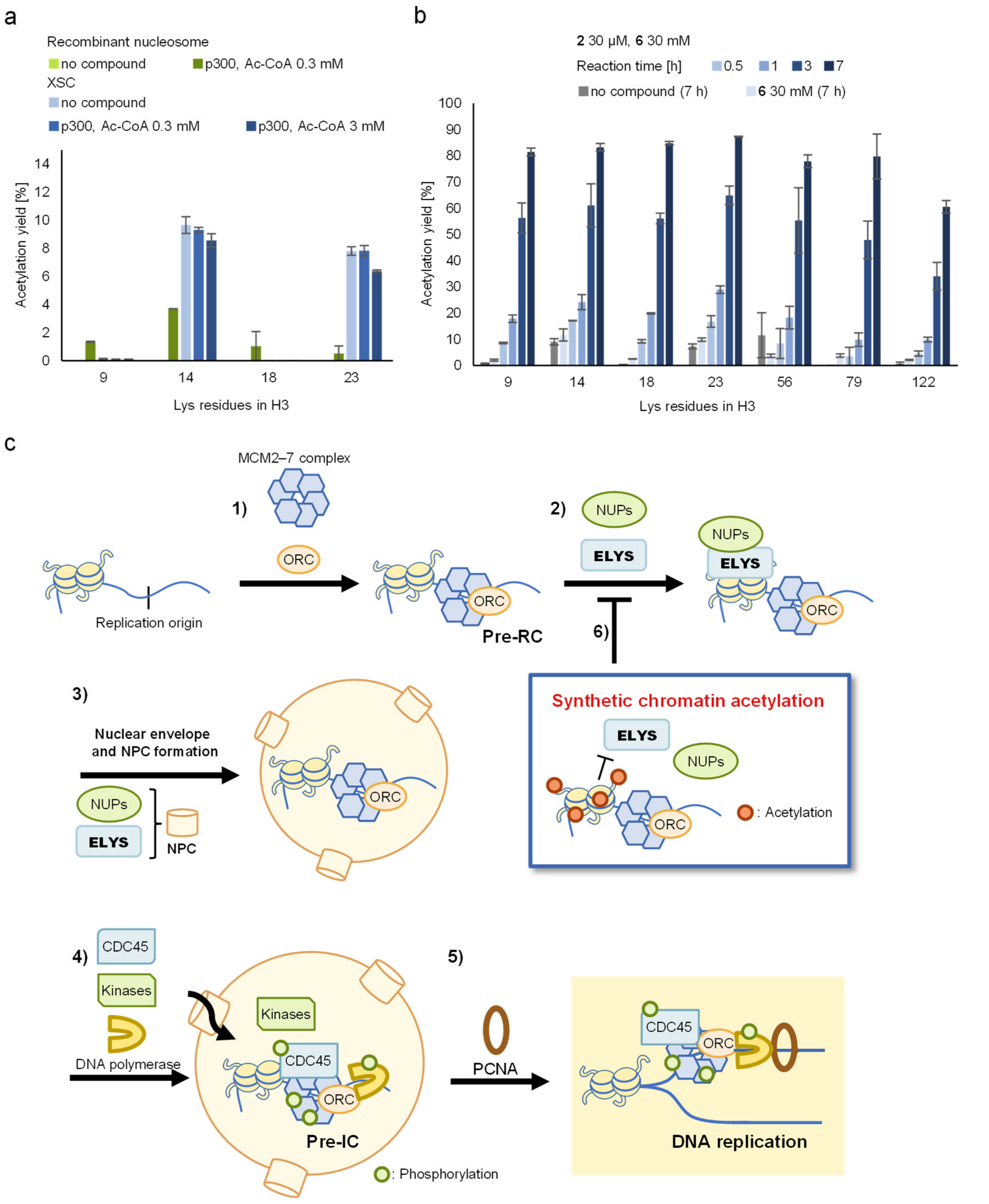

Figure S4. (a) Yield of histone acetylation of recombinant nucleosomes (0.2 µM as DNA concentration, green bars) or XSP (20000 sperm/µL, blue bars) by p300 (5 ng/µL) and Ac-CoA (0.3, 3 mM) for 5 h at 25 °C. (b) Yield of histone acetylation of XSP (20000 sperm/µL) by **2** (30 µM) and **6** (30 mM) for 0.5, 1, 3 or 7 h at 25 °C. In (a) and (b), the stoichiometry of the acetylated lysines of H3 tail and/or fold domain were analyzed by LC-MS/MS. The average and error range (bars) are indicated ((a) twice measurement in the same experiments; (b) n = 2 independent experiments). (c) Brief scheme of DNA replication.[1] 1) First, the origin

recognition complex (ORC) is recruited to replication origin. This is followed by the binding of the MCM2–7 complex, resulting in formation of pre-RC. 2) ELYS accumulates on chromatin and recruits NPC components such as NUPs. 3) Nuclear envelope and NPCs are formed. 4) CDC45, kinases and DNA polymerase transport into the nucleus via NPCs. Kinases phosphorylate MCM2–7 complex, CDC45 and DNA polymerase, and promote their loading on origin, resulting in formation of pre-IC. 5) PCNA is recruited to pre-IC and DNA replication occurs. 6) Synthetic chromatin acetylation inhibits the binding of ELYS on chromatin and consequently DNA replication downstream of it.

2. Experimental procedures

General

NMR spectra were recorded on JEOL JNM-ECX500 spectrometer, operating at 500 MHz for ^{1}H NMR and 124.51 MHz for ^{13}C NMR. Chemical shifts were reported in ppm on the δ scale relative to residual $CHCl_3$ (δ = 7.24 for ^{1}H NMR and δ = 77.0 for ^{13}C NMR) or CHD_2OD (δ = 3.31 for ^{1}H NMR and δ = 49.0 for ^{13}C NMR) as an internal reference, respectively. Analytical HPLC was conducted by using a JASCO HPLC system equipped with a UV-2075 spectrometer, PU-2080 pumps, a DG-2080-54 degasser, and an MX-2080-32 mixer or Shimadzu HPLC system equipped with an SPD-20A spectrometer, LC-20AD pumps, and a DGU-20A3R degasser. Preparative HPLC was conducted by using a JASCO HPLC system equipped with a UV-2075 spectrometer, PU-2086 pumps, a DG-2080-53 degasser, an MX-2080-32 mixer or Shimadzu HPLC system equipped with an SPD-20A spectrometer, and LC-6AD pumps. ESI-MS spectra were measured on Bruker micrOTOF II spectrometer (for HRMS). MALDI/TOF-MS was obtained with a Shimadzu Biotech Axima ToF^2 spectrometer. LC-MS/MS analyses were conducted with an AB Sciex Triple TOF 4600 equipped with an Eksigent ekspert microLC 200.

Materials

All protected α-amino acids were purchased from Watanabe Chemical Industries, Ltd. (Hiroshima, Japan) and Peptide Institute, Inc. (Osaka, Japan). Rink-Amide-AM resin was purchased from Merck KGaA, Co. NMD, **Ac-5** and PhSAc were purchased from Tokyo chemical Industry (Tokyo, Japan). **Ac-1** was purchased from Sigma-Aldrich Japan (Tokyo, Japan). Other chemicals were used as received from commercial sources (Sigma-Aldrich Japan (Tokyo, Japan), Tokyo chemical Industry (Tokyo, Japan), Kanto chemical (Tokyo, Japan) or FUJIFILM Wako Pure Chemical Corporation (Osaka, Japan)), unless otherwise stated. 8DMAP (**1**)[2], 3Py8DMAP3Py (**3**)[2], 3NMD8R (**4**)[2], **Ac-2**[3], **Ac-3**[4], **Ac-4**[5] and Ph(CN)OAc[6] were synthesized as reported.

Synthetic procedures

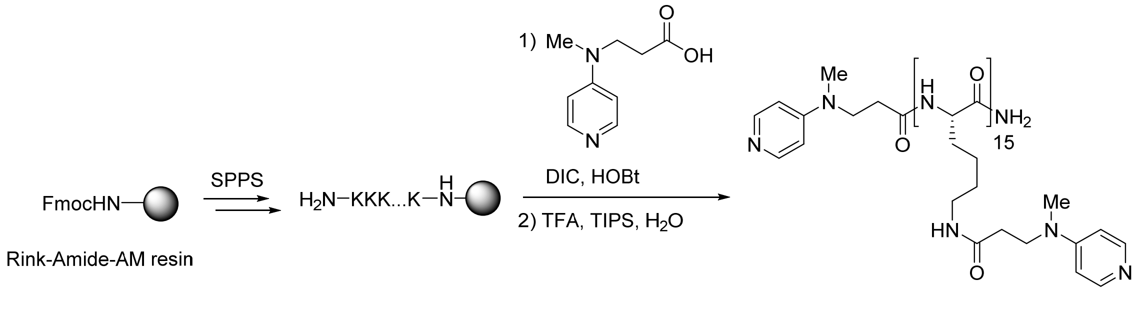

16DMAP (**2**)

16DMAP (2)

16DMAP was synthesized on a solid phase in 0.200 mmol scale using Rink-Amide-AM resin. Fmoc-Lys(Mtt)-OH (0.600 mmol, Mtt: 4-Methyltrityl) was sequentially coupled using a DIC–HOBt method (0.600 mmol each) for 60 min at room temperature after removal of each Fmoc group with 20% piperidine–DMF for 10 min to obtain pentadeca-Lys(Mtt) on resin. The Mtt groups were removed by treatment with TFA and TIPS in CH_2Cl_2 (3:5:92) for 30 sec x 12 times at room temperature. The deprotected peptide on the resin was treated with 3-(methyl-4-pyridylamino)propionic acid[7] (4.80 mmol), DIC (4.80 mmol), and HOBt (4.80 mmol) in DMF for 24 h at room temperature to afford 16DMAP on the resin. 16DMAP was cleaved from the resin by treatment with TFA in the presence of TIPS and water (95:2.5:2.5) for 60 min at room temperature, concentrated under reduced pressure, and precipitated with ether to afford crude 16DMAP, which was purified with preparative HPLC (YMC-Pack ODS-AM, 20 mm I.D. x 250 mm, linear gradient; 10–100% acetonitrile in 0.1% aqueous TFA over 40 min with a flow rate of 10 mL/min) to afford 16DMAP (**2**,133 mg (16TFA salt), 20.9 μmol, y. 10%) as white solids after lyophilization.

MALDI/TOF-MS (CHCA) *m*/*z* Calcd: 4532.73 $[M+H]^+$, Found: 4532.72; Retention time: 11.4 min (YMC-Triart C18, 4.6 mm I.D. x 150 mm, linear gradient of 0–100% acetonitrile in 0.1% aqueous TFA over 20 min with a flow rate of 0.9 mL/min). Purity: >95% (HPLC analysis at 254 nm).

Synthesis of 3PAc8R and PAc-gly

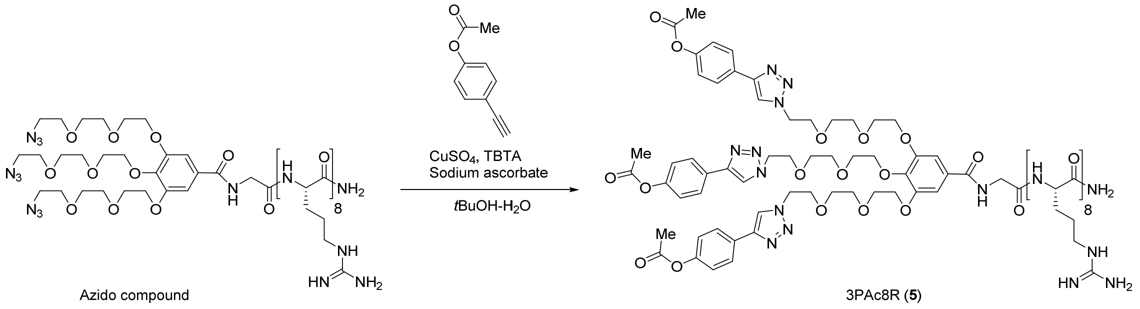

3PAc8R (5):

To a stirred solution of azido compound[2] (5.70 mg, 2.50 μmol) in water–*t*BuOH (1:1, 1.8 mL), 4-ethynylphenyl acetate[8] solution in *t*BuOH (106 mM, 400 μL, 42.4 mmol), Cu-TBTA solution in water–*t*BuOH ($CuSO_4$:TBTA = 1:2, 1.4 mL; separately prepared by mixing TBTA solution in *t*BuOH (7.42 mM, 1.08 mL, 8.01 mmol) and $CuSO_4$ aqueous solution (12.5 mM, 0.320 mL, 4 μmol)), and sodium ascorbate aqueous solution (50 mM, 0.400 mL, 20.0 μmol) were added. The mixture was stirred at room temperature for 3.5 h. Volatiles were removed under reduced pressure, and the residue was suspended in water (5 mL). Insoluble materials were removed by filtration, and filtrate was purified with preparative HPLC (YMC-Triart C18, 20 mm I.D. x 250 mm, linear gradient; 0–100% acetonitrile in 0.1% aqueous TFA over 60 min with a flow rate of 10 mL/min) to afford 3PAc8R (**5**, 4.78 mg (8TFA salt), 1.43 μmol, y. 57%) as white solids after lyophilization.

MALDI/TOF-MS (CHCA) *m*/*z* Calcd: 2427.29 $[M+H]^+$, Found: 2427.18; Retention time: 14.2 min (YMC-Triart C18, 4.6 mm I.D x 150 mm, linear gradient of 0–100% acetonitrile in 0.1% aqueous TFA over 20 min with a flow rate of 0.9 mL/min). Purity: >95% (HPLC analysis at 254 nm).

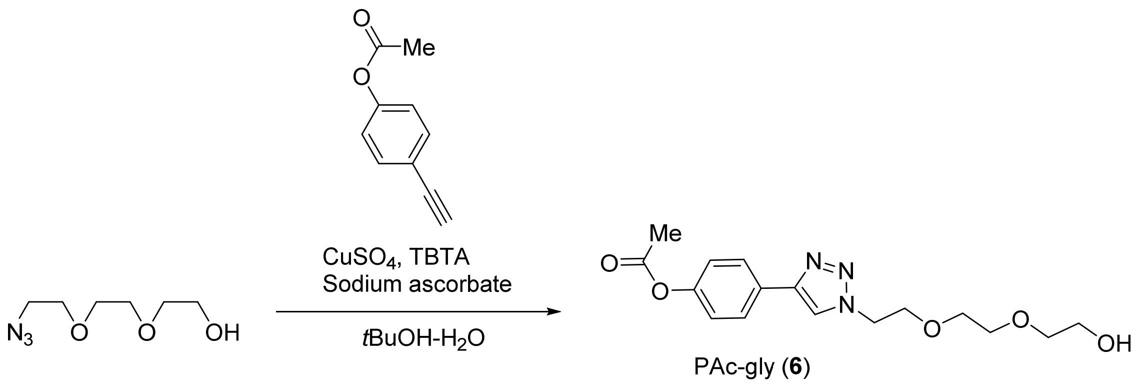

PAc-gly (**6**)

PAc-gly (6):

To the mixture of 2-(2-(2-azidoethoxy)ethoxy)ethan-1-ol[9] (69.2 mg, 0.395 mmol) and alkyne 4-ethynylphenyl acetate (95.0 mg, 0.593 mmol) in water–*t*BuOH (1:1, 6.9 mL), Cu-TBTA solution in water–*t*BuOH ($CuSO_4$:TBTA = 1:2, 7.0 mL; separately prepared by mixing TBTA solution in *t*BuOH (11.3 mM, 3.50 mL, 39.5 μmol) and $CuSO_4$ aqueous solution (5.66 mM, 3.50 mL, 19.8 μmol)), and sodium ascorbate aqueous solution (494 mM, 200 mL, 0.0988 mmol) were added. The mixture was stirred at room temperature for 1.5 h. Volatiles were removed under reduced pressure and the residue was taken up in CH_2Cl_2, and organic layer was washed with 10% $CuSO_4$ aq and brine, dried over Na_2SO_4, filtered, and concentrated to afford crude PAc-gly, which was purified with silica gel column chromatography (AcOEt/MeOH = 6/4) to afford PAc-gly (**6**, 122.9 mg, 0.366 mmol, y. 93%) as colorless oil.

^{1}H NMR ($CDCl_3$, 500 MHz) δ 7.92 (s, 1H), 7.83 (d, *J* = 8.6 Hz, 2H), 7.13 (d, *J* = 8.6 Hz, 2H), 4.57 (t, *J* = 4.6 Hz, 2H), 3.89 (t, *J* = 5.2 Hz, 2H), 3.68 (t, *J* = 4.6 Hz, 2H), 3.60–3.62 (m, 4H), 3.54 (t, *J* = 4.6 Hz, 2H), 2.29 (s, 3H); ^{13}C NMR ($CDCl_3$, 125 MHz) δ 169.4, 150.4, 146.9, 128.4, 126.8, 122.0, 121.0, 72.4, 70.6, 70.2, 69.5, 61.7, 50.4, 21.1; ESI-MS *m*/*z* 358 $[M+Na]^+$; ESI-HRMS: *m*/*z* calcd for $C_{16}H_{21}N_3O_5Na$ $[M+Na]^+$: 358.1373. Found: 358.1371.

Synthesis of X-AKFR peptide

Fmoc-Ala-Lys(Boc)-Phe-Arg(Pbf) on resin:

Fmoc-Ala-Lys(Boc)-Phe-Arg(Pbf: 2,2,4,6,7-Pentamethyldihydrobenzofuran-5-sulfonyl) on resin was synthesized on a solid phase in 0.183 mmol scale using Rink-Amide-AM resin. Fmoc-Arg(Pbf)-OH (0.549 mmol), Fmoc-Phe-OH (0.549 mmol), Fmoc-Lys(Boc)-OH (0.549 mmol), Fmoc-Ala-OH (0.549 mmol) were sequentially coupled using a DIC–HOBt method (0.549 mmol each) for 60 min at room temperature after removal of each Fmoc group with 20% piperidine–DMF for 10 min to obtain Fmoc-Ala-Lys(Boc)-Phe-Arg(Pbf) on resin.

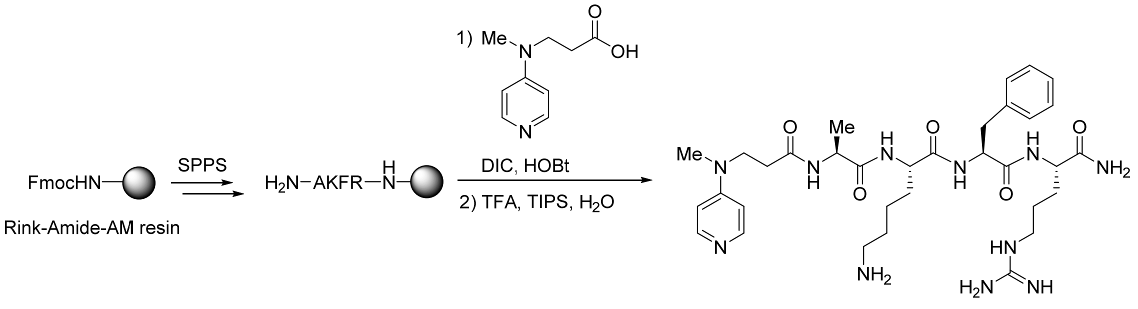

DMAP-AKFR (**7**)

DMAP-Ala-Lys-Phe-Arg-NH2 (DMAP-AKFR, 7):

Fmoc-Ala-Lys(Boc)-Phe-Arg(Pbf) on resin (0.0276 mmol) was used for the subsequent synthesis. The Fmoc group was removed by treatment with 20% piperidine–DMF for 10 min at room temperature. The Fmoc-removed peptide on the resin was treated with 3-(methyl-4-pyridylamino)propionic acid (0.0549 mmol), DIC (0.0549 mmol), and HOBt (0.0549 mmol) in DMF for 12 h at room temperature to afford DMAP-Ala-Lys(Boc)-Phe-Arg(Pbf) on the resin. The peptide was cleaved from the resin and the protecting groups were simultaneously removed by treatment with TFA in the presence of TIPS and water (95:2.5:2.5) for 60 min at room temperature, concentrated under reduced pressure, and precipitated with ether to afford crude DMAP-AKFR, which was purified with preparative HPLC (YMC-Triart C18, 20 mm I.D. x 250 mm, linear gradient; 0–100% acetonitrile in 0.1% aqueous TFA over 100 min with a flow rate of 10 mL/min) to afford DMAP-AKFR (**7**, 2.47 mg (2TFA salt), 2.71 mmol, y. 10%) as white solids after lyophilization.

MALDI/TOF-MS (CHCA) *m/z* Calcd: 682.41 $[M+H]^+$, Found: 682.64; Retention time: 10.9 min (YMC-Triart C18, 4.6 mm I.D x 150 mm, linear gradient of 0–100% acetonitrile in 0.1% aqueous TFA over 20 min with a flow rate of 0.9 mL/min). Purity: >95% (HPLC analysis at 254 nm).

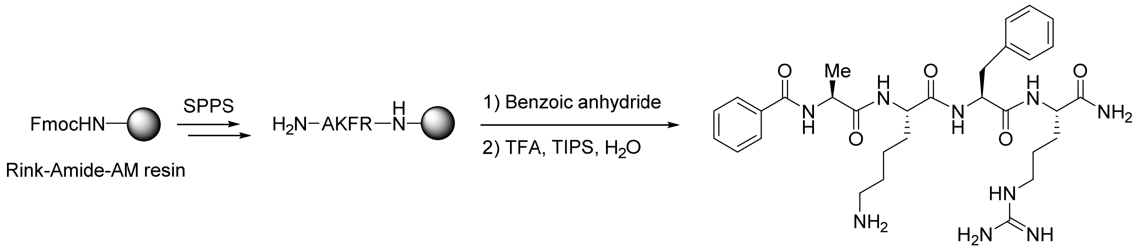

Bz-AKFR (**8**)

Bz-Ala-Lys-Phe-Arg-NH_2 (Bz-AKFR, 8):

Fmoc-Ala-Lys(Boc)-Phe-Arg(Pbf) on resin (0.0153 mmol) was used for the subsequent synthesis. The Fmoc group was removed by treatment with 20% piperidine–DMF for 10 min at room temperature. The Fmoc-removed peptide on the resin was treated with 25% benzoic anhydride–CH_2Cl_2 for 5 min at room temperature to afford Bz-Ala-Lys(Boc)-Phe-Arg(Pbf) on the resin. The peptide was cleaved from the resin and the protecting groups were simultaneously removed by treatment with TFA in the presence of TIPS and water (95:2.5:2.5) for 60 min at room temperature, concentrated under reduced pressure, and precipitated with ether to afford crude Bz-AKFR, which was purified with preparative HPLC (YMC-Triart C18, 20 mm I.D. x 250 mm, linear gradient; 0–100% acetonitrile in 0.1% aqueous TFA over 100 min with a flow rate of 10 mL/min) to afford Bz-AKFR (**8**, 1.70 mg (TFA salt), 2.30 mmol, y. 15%) as white solids after lyophilization.

MALDI/TOF-MS (CHCA) *m*/*z* Calcd: 624.36 $[M+H]^+$, Found: 624.52; Retention time: 12.6 min (YMC-Triart C18, 4.6 mm I.D x 150 mm, linear gradient of 0–100% acetonitrile in 0.1% aqueous TFA over 20 min with a flow rate of 0.9 mL/min). Purity: >95% (HPLC analysis at 254 nm).

Chemical experiments

HPLC analysis of hydrolysis ratio of 4 and 5

4 or **5** was dissolved in PBS buffer (pH 7.4) and incubated at 25 or 37 °C. Samples were analyzed after 0, 2, 4, 6 h by analytical HPLC, which was performed using YMC-Triart C18 (4.6 mm I.D. x 150 mm) column using a linear gradient of 0–100% acetonitrile in 0.1% aqueous TFA over 20 min at room temperature with a flow rate of 1 mL/min at 254 nm.

Investigation of acetyl donor-ability using 7 or 8

7 or **8** (0.5 mM) and acetyl donor (NMD, **Ac-1**–**5** ; 10 mM) were mixed in PBS buffer (pH 7.4, 20% acetonitrile), and the mixture was incubated at 30 °C. Samples were analyzed after 2, 6, 18 h by MALDI/TOF-MS. The acetylation yield was calculated by dividing the peak intensity of the acetylated mass peak with the sum of the acetylated and unacetylated mass peaks.

In vitro acetylation assay using recombinant nucleosomes

Recombinant nucleosomes (0.2 μM as DNA concentration) were mixed with cytoplasmic/nucleoplasmic proteins purified from HeLa cells, and the mixture was used as substrates. Catalyst (**1**, **2** and **3**) and/or acetyl donor (**4**, **5** and **6**) were mixed with the substrates in 20 mM Tris-HCl (pH 7.5) buffer with 150 mM potassium chloride and 1.5 mM magnesium chloride, and the whole was incubated at 25 °C. Samples were analyzed by the Western blotting using an anti-Ac-Lys antibody (9441, Cell Signaling Technology) or LC-MS/MS performed as described previously[2].

Biochemical experiments

Antibody

Mouse monoclonal antibody against PCNA PC-10 was purchased from Santa Crutz Biotechnology and used for immunoblotting. Mouse monoclonal MCM7 antibody ab2360 was purchased from Abcam. Rabbit polyclonal MCM2 antibody ab4461 was purchased from Abcam. Rabbit polyclonal histone H3 antibody ab1791 was purchased from Abcam. Mouse monoclonal nuclear pore complex proteins antibody ab24609 was purchased from Abcam. Cdc45 antibody was generous gift (Shintomi K, RIKEN).

Rabbit polyclonal antibody against *X. laevis* ELYS was raised against the peptide CRGRRGRVITSDDLRE by SCRUM Inc.

In vitro acetylation assay using XSC

Xenopus laevis sperm chromatin (XSC, 20000 sperm/μL) was used as substrates. **2** and/or **6** were mixed with the substrates in 20 mM HEPES-KOH (pH 7.5) buffer with 150 mM potassium chloride and 1.5 mM magnesium chloride, and the whole was incubated at 25 °C. Samples were analyzed by the Western blotting using an anti-Ac-Lys antibody (9441, Cell Signaling Technology) or LC-MS/MS.

***Xenopus* egg extracts**

Preparation of interphase egg extracts was performed as described previously[10] with minor modifications. Briefly, all extracts were supplemented with energy regeneration mix (2 mM ATP, 20 mM phosphocreatine, and 5 μg/mL creatine kinase). An untreated or an acetylated XSC (3,000-4,000 sperm/μL in the final reaction) was added to egg extracts and incubated at 22 °C. For chromatin spin-down from the egg extracts, sperm nuclei were incubated in 15-25 μL of the extract preparation. The extracts were diluted with 200 μL of ice-cold chromatin purification buffer (CPB; 50 mM KCl, 5 mM $MgCl_2$, 20 mM HEPES-KOH, pH 7.7) containing 2% sucrose, 0.1% NP-40, and 2 mM *N*-ethylenemaleimide (NEM) and kept on ice for 5 min. Underlay diluted extracts with 1.5 mL CPB containing 30% sucrose and centrifuged at 15,000 g for 10 min at 4 °C using a swing–bucket rotor. The pellets were resuspended in Laemmli sample buffer. The DNA replication efficiency was determined as described previously[11].

Immunofluorescence microscopy

An untreated or an acetylated XSC was incubated in the egg extracts containing 10 μM Cy3-dCTP (PA53021, GE healthcare). 2 μL extracts were diluted with 2 μL EB (10 mM HEPES-KOH, pH 7.7, 100 mM KCl, 0.1 mM $CaCl_2$, 1 mM $MgCl_2$, 50 mM sucrose) containing 25 % glycerol and 10% formalin, 5 μg/mL Hoechst and were mounted between slide and coverslip to visualize DNA and membrane vesicle. Images were acquired with AxioVision software on a Zeiss AxioObserver microscope.

Computational general information

All the calculations were carried out with Gaussian 16 (Revision B.01) program package[12]. Unless otherwise noted, geometry optimization and vibrational analysis were carried out at M06-2X[13] with 6-31+G** basis set[14]. All stationary points were optimized without any symmetry assumptions and characterized by normal coordinate analysis at the same level of theory (number of imaginary frequencies, NIMAG, 0 for minima and 1 for TSs). The intrinsic reaction coordinate (IRC) method was used to track minimum energy paths from transition structures to the corresponding local minima[15–18]. The self-consistent reaction field (SCRF) method based on the Polarizable Continuum Model (PCM)[19,20] was employed to evaluate the solvent reaction field (water; ε = 78.3553).

Cartesian coordinates and energies

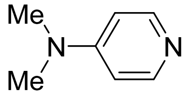

DMAP

Energy (RM062X): -382.113956571 A.U.

Gibbs Free Energy: -381.983873 A.U.

C	1.960831	1.133710	0.012193
C	0.571033	1.198667	-0.019753
C	-0.181489	0.000001	-0.047400
C	0.571013	-1.198643	-0.019781
C	1.960830	-1.133705	0.012136
N	2.677486	-0.000007	0.026820
H	2.534922	2.059183	0.030517
H	0.091718	2.170517	-0.022173
H	0.091724	-2.170508	-0.022151
H	2.534906	-2.059187	0.030480
N	-1.559204	-0.000010	-0.103169
C	-2.286199	-1.252816	0.039586
H	-2.120354	-1.725306	1.020632
H	-3.354807	-1.059711	-0.071737
H	-1.994756	-1.967348	-0.739488
C	-2.286239	1.252818	0.039539
H	-1.994778	1.967332	-0.739545
H	-3.354835	1.059675	-0.071790
H	-2.120390	1.725286	1.020576

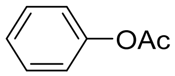

PAc

Energy (RM062X): -459.937772003 A.U.

Gibbs Free Energy: -459.849882 A.U.

C	-2.790962	0.043694	0.370770
C	-2.149884	1.139477	-0.222696
C	-0.833347	1.014265	-0.686349
C	-0.157889	-0.206730	-0.556538
C	-0.798967	-1.302512	0.036929
C	-2.115504	-1.177300	0.500583
H	-3.796167	0.139297	0.724781
H	-2.665613	2.071732	-0.321813
H	-0.343870	1.850918	-1.139475
H	-0.283239	-2.234767	0.136044
H	-2.604981	-2.013954	0.953708
O	1.185515	-0.334498	-1.029656
C	2.094961	0.006640	0.019835
O	3.231051	-0.532397	0.067886
C	1.684045	1.033681	1.091253
H	1.001116	1.739041	0.665841
H	1.212064	0.528017	1.907610
H	2.553448	1.547577	1.444735

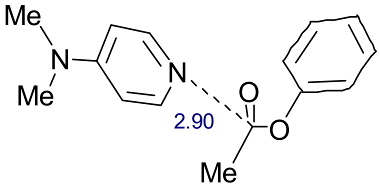

IM$_{PAc}$1

Energy (RM062X): -842.067606668 A.U.

Gibbs Free Energy: -841.823991 A.U.

C	1.845898	-1.256218	-0.305590
C	3.198594	-1.019493	-0.494467
C	3.755527	0.201883	-0.043527
C	2.856960	1.108581	0.569678
C	1.524399	0.753935	0.701744
N	0.998688	-0.404472	0.284480
H	1.409631	-2.192191	-0.652227
H	3.803757	-1.772612	-0.982784
H	3.185472	2.071280	0.939374
H	0.832706	1.449536	1.174535
N	5.076158	0.488905	-0.189262

C	5.597981	1.753427	0.299867
H	5.440463	1.858068	1.379873
H	6.667714	1.795385	0.103629
H	5.121336	2.602530	-0.203705
C	5.962578	-0.479591	-0.810960
H	5.663668	-0.687881	-1.844925
H	6.974295	-0.078300	-0.820886
H	5.971688	-1.424747	-0.255461
C	-5.866308	1.095168	-0.294834
C	-4.831117	2.030518	-0.357130
C	-3.501684	1.617298	-0.327490
C	-3.213083	0.255082	-0.247110
C	-4.234581	-0.692378	-0.185355
C	-5.561233	-0.261648	-0.202613
H	-6.900418	1.421221	-0.323410
H	-5.058186	3.089432	-0.430439
H	-2.685026	2.330154	-0.375310
H	-4.001529	-1.752169	-0.145960
H	-6.356314	-0.999001	-0.159258
O	-1.883679	-0.105670	-0.306955
C	-1.511364	-1.321510	0.250671
O	-1.262390	-2.247872	-0.471822
C	-1.504963	-1.350372	1.750239
H	-0.906553	-2.197097	2.084624
H	-1.094713	-0.417872	2.137835
H	-2.528983	-1.466530	2.119434

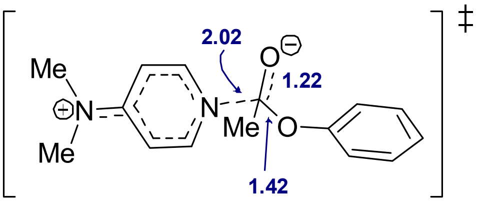

TS$_{PAc}$1

Energy (RM062X): -842.062580376 A.U.

Gibbs Free Energy: -841.804158 A.U.

C	1.567025	-1.317860	-0.476129
C	2.902282	-1.013173	-0.646484
C	3.437606	0.173946	-0.068958
C	2.518595	0.985551	0.659274
C	1.200339	0.597644	0.776290
N	0.729070	-0.536159	0.221984
H	1.111039	-2.205362	-0.904481
H	3.518405	-1.690077	-1.222396
H	2.826723	1.911877	1.124537
H	0.483441	1.206325	1.315218
N	4.743278	0.514452	-0.204709
C	5.254305	1.743352	0.403028
H	5.108932	1.739448	1.489166
H	6.321911	1.818542	0.203861
H	4.763504	2.630570	-0.014093
C	5.649096	-0.333297	-0.980031
H	5.318808	-0.421656	-2.021530
H	6.642298	0.112193	-0.974110
H	5.721358	-1.338175	-0.548363
C	-5.654001	0.878661	-0.444854
C	-4.721167	1.923391	-0.454522
C	-3.359994	1.658835	-0.305898
C	-2.905228	0.337481	-0.147353
C	-3.834643	-0.715781	-0.146549
C	-5.198286	-0.433029	-0.290010
H	-6.713926	1.084688	-0.558205
H	-5.053206	2.950960	-0.575056
H	-2.631974	2.464507	-0.308334
H	-3.497781	-1.739880	-0.052822
H	-5.907055	-1.256872	-0.285536
O	-1.549358	0.196576	-0.015420
C	-0.969793	-1.118182	0.344914
O	-1.124725	-2.055863	-0.464694
C	-1.121796	-1.357689	1.846348
H	-0.502594	-2.210074	2.135972
H	-0.842846	-0.486881	2.443275

H	-2.166838	-1.602988	2.058488

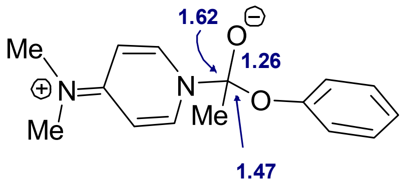

IM$_{PAc}$2

Energy (RM062X): -842.065803712 A.U.

Gibbs Free Energy: -841.803255 A.U.

C	1.552824	-1.383613	-0.396241
C	2.887663	-1.083471	-0.520099
C	3.375697	0.170615	-0.061144
C	2.415547	1.053091	0.511951
C	1.101587	0.668527	0.599400
N	0.676515	-0.528788	0.151882
H	1.116079	-2.316728	-0.735023
H	3.541670	-1.816020	-0.972127
H	2.692392	2.030308	0.882626
H	0.342222	1.318427	1.018114
N	4.673675	0.507368	-0.163597
C	5.132904	1.807895	0.308930
H	4.944915	1.925111	1.381069
H	6.203953	1.885650	0.136369
H	4.634814	2.619836	-0.230774
C	5.623682	-0.426165	-0.756069
H	5.358265	-0.650365	-1.794402
H	6.613897	0.023432	-0.741952
H	5.659045	-1.361686	-0.188525
C	-5.608682	0.776320	-0.326384
C	-4.717486	1.848587	-0.410655
C	-3.344938	1.631575	-0.345829
C	-2.838657	0.331970	-0.201389
C	-3.726223	-0.750004	-0.127081
C	-5.101063	-0.513738	-0.183885
H	-6.679404	0.944790	-0.374310
H	-5.091612	2.861799	-0.523437
H	-2.643595	2.458241	-0.405054
H	-3.340411	-1.758049	-0.044371
H	-5.779912	-1.359467	-0.122831
O	-1.481920	0.230199	-0.166712
C	-0.865726	-1.031807	0.292140
O	-1.064407	-2.049592	-0.435075
C	-1.088495	-1.171148	1.801590
H	-0.457765	-1.984264	2.168205
H	-0.854152	-0.254923	2.348822
H	-2.132929	-1.432818	1.982489

$^{\oplus}NH_4Me$

$^{+}NH_4Me$

Energy (RM062X): -96.2665286844 A.U.

Gibbs Free Energy: -96.211208 A.U.

C	0.000000	0.000000	-0.770000
H	0.000000	-1.008806	-1.126667
H	-0.873651	0.504403	-1.126667
H	0.873651	0.504403	-1.126667
N	0.000000	0.000000	0.700000
H	0.000000	0.942809	1.033333
H	-0.816497	-0.471405	1.033333
H	0.816497	-0.471405	1.033333

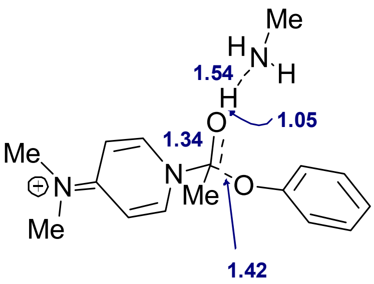

IM$_{PAc}$4

Energy (RM062X): -938.365915743 A.U.

Gibbs Free Energy: -938.027927 A.U.

C	-1.698842	0.911396	0.928831
C	-3.012221	0.974539	0.552412
C	-3.609900	-0.112202	-0.150789
C	-2.768638	-1.235267	-0.409650
C	-1.464859	-1.225254	-0.001509
N	-0.926884	-0.163940	0.647760
H	-1.208453	1.713999	1.464211
H	-3.571867	1.864263	0.804331
H	-3.133170	-2.110953	-0.927627
H	-0.800030	-2.058833	-0.187906
N	-4.887745	-0.083138	-0.543054
C	-5.464349	-1.221645	-1.254140
H	-6.500680	-0.996187	-1.493032
H	-4.925137	-1.410088	-2.187051
H	-5.436760	-2.122432	-0.633668
C	-5.718571	1.081993	-0.250638
H	-6.714552	0.911168	-0.651852
H	-5.799695	1.240827	0.828800
H	-5.304040	1.980711	-0.716858
C	5.252687	-0.653344	-0.725823
C	4.327639	-1.055627	-1.692352
C	4.800683	-0.304459	0.543958
C	2.971368	-1.103769	-1.389324
H	4.660699	-1.327963	-2.688788
C	3.441615	-0.345987	0.864987
H	5.506526	0.015267	1.304124
C	2.529121	-0.749600	-0.111267
H	2.237484	-1.405766	-2.129588
H	3.112810	-0.032457	1.846268
O	1.166397	-0.839416	0.059678
C	0.516331	-0.209845	1.154302
C	0.539286	-1.069405	2.406950
H	0.128343	-2.058309	2.194609
H	1.563285	-1.180935	2.764416
H	-0.058605	-0.585876	3.181857
O	0.950336	1.041749	1.389470
C	2.848775	2.636930	-0.993830
H	3.450577	1.727797	-1.050876
H	3.163837	3.203674	-0.115682
N	1.428199	2.266702	-0.840230
H	1.135428	1.686564	-1.624884
H	1.138026	1.546786	0.477285
H	0.848555	3.102933	-0.883813
H	3.027530	3.239166	-1.888806
H	6.310300	-0.610109	-0.962684

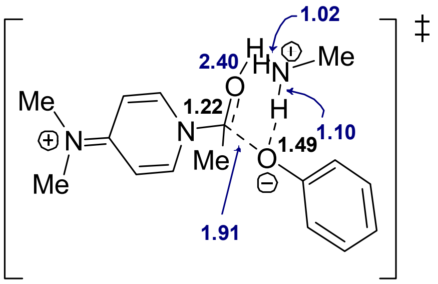

TS$_{PAc}$4

Energy (RM062X): -938.336294974 A.U.

Gibbs Free Energy: -938.011784 A.U.

C	-1.757220	0.858314	1.175610
C	-3.073471	0.741042	0.831359
C	-3.503941	-0.318376	-0.025191
C	-2.483746	-1.206238	-0.486942
C	-1.185666	-1.034339	-0.099531
N	-0.812385	-0.013655	0.726144
H	-1.396762	1.638675	1.832477
H	-3.770565	1.458309	1.241359
H	-2.708533	-2.025585	-1.155783
H	-0.397552	-1.680788	-0.455348
N	-4.790890	-0.469243	-0.375293
C	-5.199760	-1.582104	-1.238536
H	-6.282710	-1.561828	-1.350815
H	-4.745227	-1.498211	-2.232376
H	-4.914729	-2.543536	-0.798881

C	-5.793157	0.506179	0.064625
H	-6.755519	0.247626	-0.373928
H	-5.893739	0.502321	1.155378
H	-5.523088	1.515628	-0.262075
C	5.505866	-0.912878	-0.258747
C	4.591928	-1.903933	-0.641968
C	5.001171	0.352917	0.075269
C	3.225276	-1.639428	-0.710377
H	4.956205	-2.898725	-0.887936
C	3.636662	0.635402	0.006310
H	5.688694	1.135839	0.389697
C	2.738691	-0.363052	-0.404143
H	2.517003	-2.410956	-0.994695
H	3.260657	1.618184	0.278497
O	1.396623	-0.140328	-0.481620
C	0.592633	0.145432	1.254102
C	1.089328	-1.110142	1.936165
H	0.920302	-2.022799	1.364338
H	2.154658	-1.003148	2.146578
H	0.549356	-1.178227	2.887525
O	0.901039	1.258798	1.686210
C	1.250266	2.976107	-1.708669
H	2.275796	3.051117	-1.340700
H	0.798883	3.971694	-1.731277
N	0.493107	2.060646	-0.819566
H	0.998987	0.969671	-0.765932
H	0.509385	2.389056	0.148401
H	-0.484128	2.006655	-1.112125
H	1.264819	2.560741	-2.719691
H	6.558536	-1.103122	-0.234421

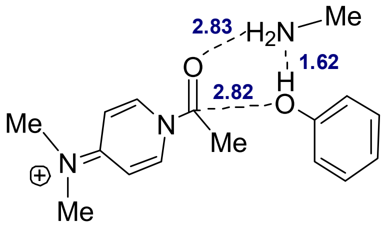

IM*PAc*5

Energy (RM062X): -938.363555341 A.U.

Gibbs Free Energy: -938.031472 A.U.

C	-1.664850	0.267582	1.541730
C	-2.893575	0.491414	1.006616
C	-3.352470	-0.296217	-0.099405
C	-2.455998	-1.306864	-0.574902
C	-1.242030	-1.479252	0.011853
N	-0.834556	-0.706988	1.062450
H	-1.277826	0.837217	2.376972
H	-3.506221	1.267752	1.442631
H	-2.712723	-1.945702	-1.407654
H	-0.544837	-2.222950	-0.346918
N	-4.543351	-0.100088	-0.652300
C	-4.985129	-0.931988	-1.773520
H	-5.986833	-0.624537	-2.061478
H	-4.318650	-0.806619	-2.631109
H	-5.012017	-1.985063	-1.481935
C	-5.428715	0.952618	-0.149956
H	-6.334333	0.961531	-0.750403
H	-5.699454	0.762365	0.891964
H	-4.945697	1.929888	-0.229785
C	5.386022	-0.349933	-0.504294
C	4.667308	-1.125549	-1.416728
C	4.705242	0.578997	0.282684
C	3.289501	-0.971623	-1.547963
H	5.182634	-1.855077	-2.034366
C	3.324253	0.736290	0.167143
H	5.248590	1.184317	1.002146
C	2.609090	-0.034905	-0.759507

H	2.722161	-1.566817	-2.257403
H	2.797107	1.441464	0.803140
O	1.264672	0.059182	-0.894923
C	0.485913	-0.843880	1.660541
C	1.227368	-2.114656	1.386833
H	1.506900	-2.178811	0.332303
H	2.132175	-2.101509	1.992048
H	0.615470	-2.981724	1.647750
O	0.875058	0.048734	2.367480
C	0.969908	3.405958	-0.766751
H	2.049983	3.351608	-0.614503
H	0.627612	4.411320	-0.505611
N	0.316321	2.350142	0.028273
H	0.902438	0.927625	-0.489833
H	0.480999	2.504497	1.021422
H	-0.691818	2.399951	-0.104019
H	0.766845	3.226866	-1.824192
H	6.459740	-0.470320	-0.406814

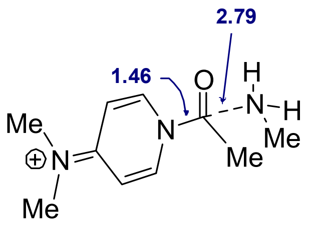

IM*PAc*6

Energy (RM062X): -630.981336932 A.U.

Gibbs Free Energy: -630.747405 A.U.

C	-0.210568	-1.159816	-0.603410
C	1.144275	-1.023355	-0.751797
C	1.850563	-0.076861	0.061108
C	1.062893	0.686609	0.981035
C	-0.273578	0.457840	1.102664
N	-0.913865	-0.475231	0.342088
H	-0.791145	-1.858519	-1.191496
H	1.641141	-1.614909	-1.507223

H	1.506589	1.440235	1.615690
H	-0.869907	1.001064	1.822406
N	3.167898	0.081225	-0.029123
C	3.854708	1.061226	0.813211
H	4.914464	1.045895	0.573391
H	3.469184	2.066642	0.625443
H	3.730021	0.814793	1.871382
C	3.947714	-0.744789	-0.951628
H	4.997536	-0.480617	-0.854979
H	3.829769	-1.804602	-0.709935
H	3.635534	-0.569593	-1.984556
C	-2.317100	-0.847402	0.551457
C	-3.100854	-0.029603	1.530850
H	-2.949432	1.041001	1.401871
H	-4.151734	-0.266256	1.372272
H	-2.823648	-0.318526	2.549672
O	-2.707231	-1.860547	0.034401
C	-2.165052	2.099221	-1.308096
H	-2.322227	2.606107	-0.350551
H	-2.428566	2.800019	-2.108977
N	-2.925901	0.850220	-1.325299
H	-3.924141	1.035920	-1.282503
H	-2.762344	0.343556	-2.191130
H	-1.098175	1.870253	-1.392019

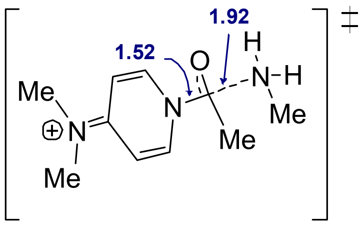

TS*PAc*6

Energy (RM062X): -630.976278062 A.U.

Gibbs Free Energy: -630.735546 A.U.

C	-0.117633	1.313271	0.151923
C	1.244416	1.233445	0.281175

C	1.933500	0.029101	-0.058328
C	1.115522	-1.042616	-0.530886
C	-0.243212	-0.889033	-0.628378
N	-0.869011	0.269862	-0.286828
H	-0.678684	2.211686	0.375788
H	1.773440	2.106792	0.636103
H	1.539531	-1.992981	-0.822589
H	-0.861688	-1.697561	-0.989652
N	3.269176	-0.087406	0.054393
C	3.941635	-1.337503	-0.314289
H	5.013085	-1.215532	-0.170085
H	3.600661	-2.167917	0.312973
H	3.761931	-1.586077	-1.365248
C	4.070965	1.036192	0.549853
H	5.113417	0.728774	0.598670
H	3.996373	1.898474	-0.121418
H	3.751653	1.335684	1.553405
C	-2.422123	0.518448	-0.407982
C	-3.028991	-0.350387	-1.505600
H	-2.866012	-1.423857	-1.399636
H	-4.105250	-0.164541	-1.511953
H	-2.618361	-0.024398	-2.465015
O	-2.720955	1.733144	-0.345834
C	-2.691830	-1.587939	1.522711
H	-3.026437	-2.276820	0.747336
H	-3.231783	-1.802217	2.447381
N	-2.969588	-0.191757	1.096249
H	-3.977425	-0.032279	1.046268
H	-2.621476	0.472131	1.791094
H	-1.622870	-1.707278	1.695365

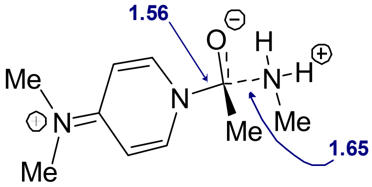

IM$_{PAc}$7

Energy (RM062X): -630.977425548 A.U.

Gibbs Free Energy: -630.735424 A.U.

C	-0.198549	1.308126	-0.044156
C	1.153983	1.256541	0.124401
C	1.866152	0.043597	-0.117962
C	1.085348	-1.067703	-0.550939
C	-0.270459	-0.943150	-0.676688
N	-0.915987	0.218405	-0.414874
H	-0.783729	2.206389	0.113350
H	1.664549	2.157787	0.434486
H	1.533591	-2.023058	-0.784167
H	-0.873153	-1.781302	-0.999554
N	3.189790	-0.044100	0.043783
C	3.878703	-1.305784	-0.216537
H	4.936192	-1.178345	0.000889
H	3.485561	-2.100948	0.423469
H	3.770275	-1.599657	-1.264973
C	3.956973	1.122078	0.474576
H	5.007376	0.847602	0.532586
H	3.850410	1.942532	-0.241130
H	3.629041	1.459854	1.462173
C	-2.459166	0.373550	-0.448707
C	-3.104898	-0.736893	-1.267117
H	-2.971171	-1.740867	-0.860354
H	-4.172540	-0.515094	-1.301531
H	-2.713598	-0.706814	-2.287267
O	-2.827055	1.558784	-0.614403
C	-2.157874	-1.169088	1.883118
H	-2.324852	-2.090137	1.324304
H	-2.578635	-1.277091	2.883559

N	-2.814360	-0.040678	1.183708
H	-3.829654	-0.163443	1.191218
H	-2.640897	0.838565	1.677079
H	-1.089707	-0.969276	1.962839

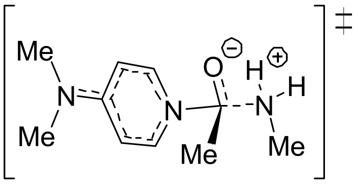

TS$_{PAc}$7

Energy (RM062X): -630.971070413 A.U.

Gibbs Free Energy: -630.729587 A.U.

C	-0.080066	1.208109	0.100549
C	1.291359	1.204849	0.209545
C	2.010924	0.001872	-0.026951
C	1.233651	-1.137177	-0.376512
C	-0.135841	-1.032503	-0.451160
N	-0.793859	0.115065	-0.205313
H	-0.660894	2.116272	0.243383
H	1.795206	2.127577	0.462949
H	1.691055	-2.093203	-0.591480
H	-0.730364	-1.898645	-0.722829
N	3.352594	-0.055304	0.065313
C	4.051478	-1.305238	-0.206509
H	5.119380	-1.146767	-0.074012
H	3.730217	-2.092285	0.483369
H	3.875257	-1.639628	-1.234207
C	4.110362	1.139048	0.417160
H	5.167664	0.886351	0.455362
H	3.968575	1.929419	-0.327490
H	3.809172	1.518544	1.398865
C	-2.643851	0.496412	-0.410503
C	-3.061216	-0.500498	-1.458916
H	-2.769312	-1.526199	-1.237669
H	-4.150433	-0.457232	-1.550507
H	-2.615276	-0.190461	-2.404494
O	-2.753429	1.709485	-0.536436
C	-2.977689	-1.378346	1.460787
H	-3.631843	-1.976174	0.829554
H	-3.299712	-1.452835	2.498183
N	-3.067941	0.045492	1.044862
H	-4.035709	0.372191	1.135367
H	-2.521185	0.638624	1.676090
H	-1.944585	-1.707362	1.372092

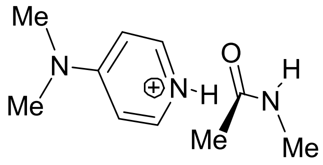

IM$_{PAc}$8

Energy (RM062X): -630.969440536 A.U.

Gibbs Free Energy: -630.772454 A.U.

C	-0.022173	1.119603	0.154965
C	1.355278	1.167021	0.225195
C	2.109154	-0.013749	-0.003927
C	1.363928	-1.181817	-0.305684
C	-0.014567	-1.113082	-0.338026
N	-0.719431	0.004437	-0.103919
H	-0.609799	2.024122	0.306946
H	1.836807	2.110544	0.445927
H	1.846292	-2.127807	-0.512815
H	-0.583595	-2.010611	-0.572489
N	3.460899	-0.023229	0.054008
C	4.190410	-1.253733	-0.213404
H	5.256399	-1.062087	-0.108970
H	3.910543	-2.040251	0.495774

H	4.001365	-1.614732	-1.230640
C	4.184623	1.203886	0.350386
H	5.251457	0.990038	0.362408
H	3.993779	1.971203	-0.408290
H	3.901284	1.601657	1.330836
C	-2.917986	0.554780	-0.422328
C	-3.144660	-0.447383	-1.505002
H	-2.737762	-1.428179	-1.266093
H	-4.224772	-0.538018	-1.664575
H	-2.679489	-0.063669	-2.412169
O	-2.852460	1.747433	-0.525659
C	-3.199228	-1.380611	1.328259
H	-3.933785	-1.896092	0.713991
H	-3.446560	-1.498926	2.381355
N	-3.234874	0.074488	1.012839
H	-4.164876	0.452827	1.229844
H	-2.577575	0.581368	1.614405
H	-2.194856	-1.750211	1.136733

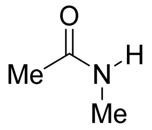

MeNHAc

Energy (RM062X): -248.431090051 A.U.

Gibbs Free Energy: -248.359223 A.U.

C	0.614234	-0.154679	-0.012207
C	0.729118	1.348724	-0.018174
H	0.323933	1.760141	-0.947590
H	0.165475	1.783053	0.812176
H	1.779741	1.618063	0.071501
O	1.590497	-0.892942	0.070566
C	-1.875102	0.076771	0.073979
H	-1.990498	0.345126	1.128453
H	-2.720266	-0.540601	-0.229303
N	-0.646220	-0.680277	-0.169083
H	-0.685410	-1.670759	0.051919
H	-1.882913	0.985553	-0.529694

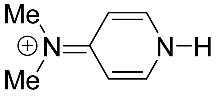

IM$_{PAc}$9

Energy (RM062X): -382.556798067 A.U.

Gibbs Free Energy: -382.427352 A.U.

C	-0.000001	1.887703	1.171067
C	0.000001	0.486455	1.200564
C	0.000003	-0.223751	0.000000
C	0.000001	0.486455	-1.200564
C	-0.000001	1.887703	-1.171067
N	0.000000	2.547770	0.000000
H	-0.000003	2.437179	2.089204
H	0.000002	-0.033820	2.135559
H	0.000002	-0.033820	-2.135559
H	-0.000003	2.437179	-2.089204
N	0.000000	-1.561451	0.000000
C	-0.000001	-2.296451	-1.273057
H	1.008804	-2.474786	-1.581940
H	-0.504406	-3.231388	-1.145114
H	-0.504403	-1.718179	-2.018765
C	-0.000001	-2.296451	1.273057
H	-0.504403	-1.718179	2.018765
H	-0.504406	-3.231388	1.145114
H	1.008804	-2.474786	1.581940
H	0.000003	3.547770	0.000000

3. References

(1) Fragkos, M.; Ganier, O.; Coulombe, P.; Méchali, M. DNA Replication Origin Activation in Space and Time. *Nat. Rev. Mol. Cell Biol.* **2015**, *16*, 360–374.

(2) Ishiguro, T.; Amamoto, Y.; Tanabe, K.; Liu, J.; Kajino, H.; Fujimura, A.; Aoi, Y.; Osakabe, A.; Horikoshi, N.; Kurumizaka, H.; Yamatsugu, K.; Kawashima, S. A.; Kanai, M. Synthetic Chromatin Acylation by an Artificial Catalyst System. *Chem* **2017**, *2*, 840–859.

(3) Haider, A.; Cornuz, G.; Wyler, H. Synthesis of 4-Oxo-1,2,3,4-Tetrahydropyridine (2,3-Dihydro-4(1H)Pyridinone). *Helv. Chim. Acta* **1975**, *58*, 1287–1292.

(4) Stübs, G.; Rupp, B.; Schumann, R. R.; Schröder, N. W. J.; Rademann, J. Chemoenzymatic Synthesis of a Glycolipid Library and Elucidation of the Antigenic Epitope for Construction of a Vaccine against Lyme Disease. *Chem. Eur. J.* **2010**, *16*, 3536–3544.

(5) Fife, T. H.; Natarajan, R.; Werner, M. H. Effect of the Leaving Group in the Hydrolysis of N-Acylimidazoles. The Hydroxide Ion, Water, and General-Base Catalyzed Hydrolysis of N-Acyl-4 (5)-Nitroimidazoles. *J. Org. Chem.* **1987**, *52*, 740–746.

(6) O'Brien, N. J.; Amran, S.; Medan, J.; Cleary, B.; Deady, L. W.; Jennings, I. G.; Thompson, P. E.; Abbott, B. M. Potent Inhibitors of Phosphatidylinositol3 (PI3) Kinase That Have Antiproliferative Activity Only When Delivered as Prodrug Forms. *ChemMedChem* **2013**, *8*, 914–918.

(7) Bhattacharya, S.; Snehalatha, K. Dialkylaminopyridine Catalysed Esterolysis of P-Nitrophenyl Alkanoates in Different Cationic Microemulsions. *J. Chem. Soc. Perkin Trans. 2* **1996**, *9*, 2021–2025.

(8) Tassano, E.; Alama, A.; Basso, A.; Dondo, G.; Galatini, A.; Riva, R.; Banfi, L. Conjugation of Hydroxytyrosol with Other Natural Phenolic Fragments: From Waste to Antioxidants and Antitumour Compounds. *European J. Org. Chem.* **2015**, *2015*, 6710–6726.

(9) Amaral, S. P.; Fernandez-Villamarin, M.; Correa, J.; Riguera, R.; Fernandez-Megia, E. Efficient Multigram Synthesis of the Repeating Unit of Gallic Acid-Triethylene Glycol Dendrimers. *Org. Lett.* **2011**, *13*, 4522–4525.

(10) Nishiyama, A.; Yamaguchi, L.; Sharif, J.; Johmura, Y.; Kawamura, T.; Nakanishi, K.; Shimamura, S.; Arita, K.; Kodama, T.; Ishikawa, F.; Koseki, H.; Nakanishi, M. Uhrf1-Dependent H3K23 Ubiquitylation Couples Maintenance DNA Methylation and Replication. *Nature* **2013**, *502*, 249–253.

(11) Lutzmann, M.; Méchali, M. MCM9 Binds Cdt1 and Is Required for the Assembly of Prereplication Complexes. *Mol. Cell* **2008**, *31*, 190–200.

(12) Gaussian 16, Revision B.01, Frisch, M. J.; Trucks, G. W.; Schlegel, H. B.; Scuseria, G. E.; Robb, M. A.; Cheeseman, J. R.; Scalmani, G.; Barone, V.; Petersson, G. A.; Nakatsuji, H.; Li, X.; Caricato, M.; Marenich, A. V.; Bloino, J.; Janesko, B. G.; Gomperts, R.; Mennucci, B.; Hratchian, H. P.; Ortiz, J. V.; Izmaylov, A. F.; Sonnenberg, J. L.; Williams-Young, D.; Ding, F.; Lipparini, F.; Egidi, F.; Goings, J.; Peng, B.; Petrone, A.; Henderson, T.; Ranasinghe, D.; Zakrzewski, V. G.; Gao, J.; Rega, N.; Zheng, G.; Liang, W.; Hada, M.; Ehara, M.; Toyota, K.; Fukuda, R.; Hasegawa, J.; Ishida, M.; Nakajima, T.; Honda, Y.; Kitao, O.; Nakai, H.; Vreven, T.; Throsse II, K.; Montgomery, J. A., Jr.; Peralta, J. E.; Ogliaro, F.; Bearpark, M. J.; Heyd, J. J.; Brothers, E. N.; Kudin, K. N.; Staroverov, V. N.; Keith, T. A.; Kobayashi, R.; Normand, J.; Raghavachari, K.; Rendell, A. P.; Burant, J. C.; Iyengar, S. S.; Tomasi, J.; Cossi, M.; Millam, J. M.; Klene, M.; Adamo, C.; Cammi, R.; Ochterski, J. W.; Martin, R. L.;

Morokuma, K.; Farkas, O.; Foresman, J. B.; Fox, D. J. Gaussian, Inc., Wallingford CT, **2016**.

(13) Zhao, Y.; Truhlar, D. G. The M06 Suite of Density Functionals for Main Group Thermochemistry, Thermochemical Kinetics, Noncovalent Interactions, Excited States, and Transition Elements: Two New Functionals and Systematic Testing of Four M06-Class Functionals and 12 Other Function. *Theor. Chem. Acc.* **2008**, *120*, 215–241.

(14) Frisch, M. J.; Pople, J. A.; Binkley, J. S. Self-Consistent Molecular Orbital Methods 25. Supplementary Functions for Gaussian Basis Sets. *J. Chem. Phys.* **1984**, *80*, 3265–3269.

(15) Fukui, K. The Path of Chemical Reactions - The IRC Approach. *Acc. Chem. Res.* **1981**, *14*, 363–368.

(16) Ishida, K.; Morokuma, K.; Komornicki, A. The Intrinsic Reaction Coordinate. An Ab Initio Calculation for HNC → HCN and H^- + CH_4 → CH_4+H^-. *J. Chem. Phys.* **1977**, *66*, 2153–2156.

(17) Gonzalez, C.; Bernhard Schlegel, H. An Improved Algorithm for Reaction Path Following. *J. Chem. Phys.* **1989**, *90*, 2154–2161.

(18) Gonzalez, C.; Schlegel, H. B. Reaction Path Following in Mass-Weighted Internal Coordinates. *J. Phys. Chem.* **1990**, *94*, 5523–5527.

(19) Tomasi, J.; Mennucci, B.; Cammi, R. Quantum Mechanical Continuum Solvation Models. *Chem. Rev.* **2005**, *105*, 2999–3093.

(20) Miertuš, S.; Scrocco, E.; Tomasi, J. Electrostatic Interaction of a Solute with a Continuum. A Direct Utilizaion of AB Initio Molecular Potentials for the Prevision of Solvent Effects. *Chem. Phys.* **1981**, *55*, 117–129.

4. Spectral data

^{1}H NMR

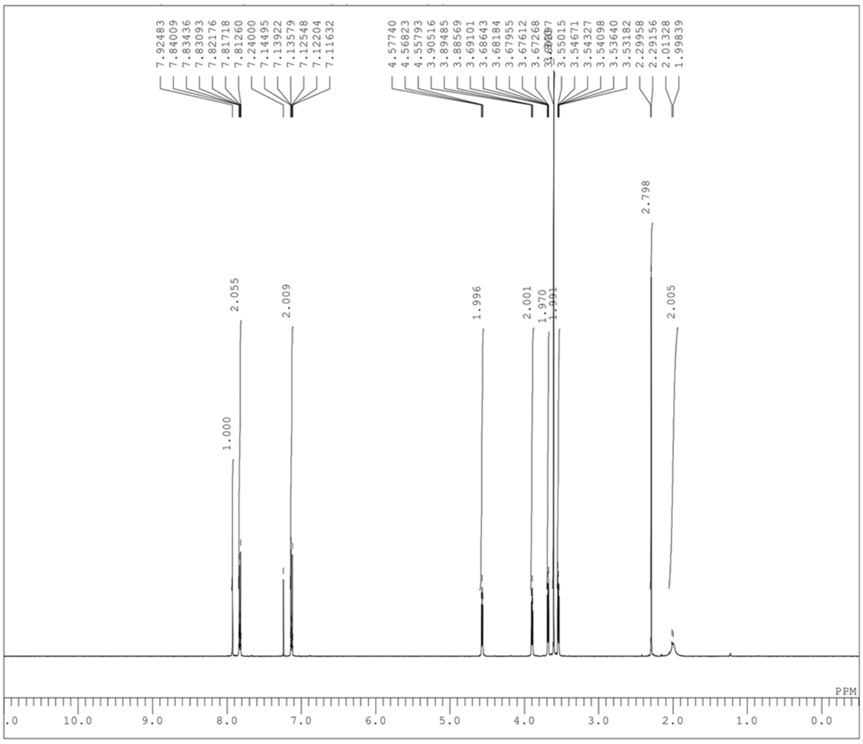

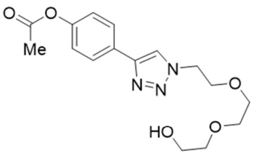

PAc-gly (**6**)

^{13}C NMR

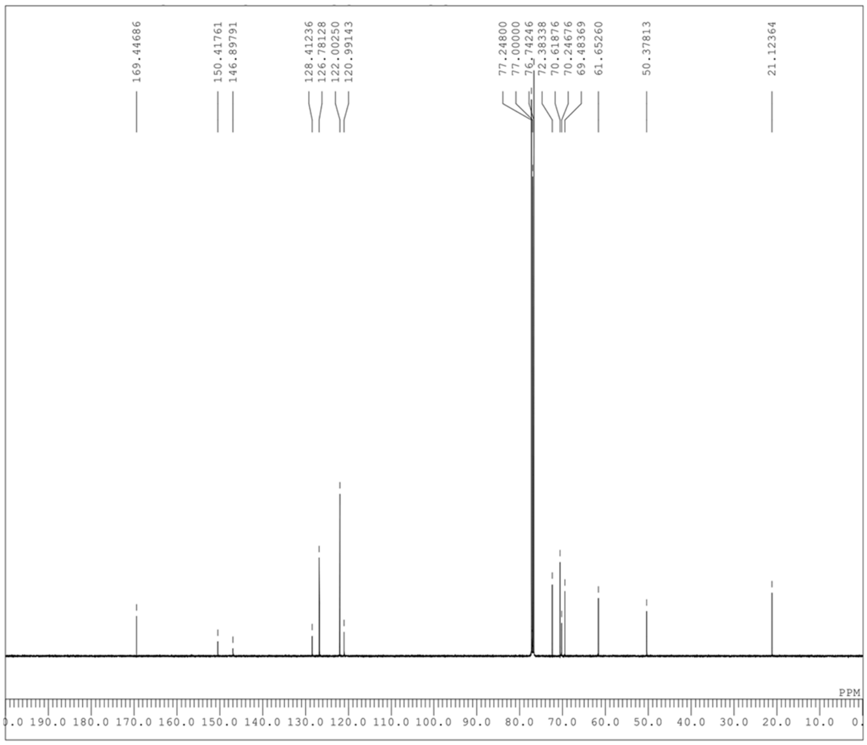

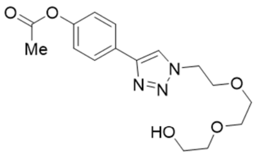

PAc-gly (**6**)
